# Scientific Validation of Human Neurosphere Assays for Developmental Neurotoxicity Evaluation

**DOI:** 10.3389/ftox.2022.816370

**Published:** 2022-03-02

**Authors:** Katharina Koch, Kristina Bartmann, Julia Hartmann, Julia Kapr, Jördis Klose, Eliška Kuchovská, Melanie Pahl, Kevin Schlüppmann, Etta Zühr, Ellen Fritsche

**Affiliations:** ^1^ IUF—Leibniz Research Institute for Environmental Medicine, Duesseldorf, Germany; ^2^ Medical Faculty, Heinrich-Heine-University, Duesseldorf, Germany

**Keywords:** developmental neurotoxicity, neural progenitor cells, neurons, oligodendrocytes, new approach methodologies, 3D *in vitro* models, human induced pluripotent stem cells, radial glia cells

## Abstract

There is a call for a paradigm shift in developmental neurotoxicity (DNT) evaluation, which demands the implementation of faster, more cost-efficient, and human-relevant test systems than current *in vivo* guideline studies. Under the umbrella of the Organisation for Economic Co-operation and Development (OECD), a guidance document is currently being prepared that instructs on the regulatory use of a DNT *in vitro* battery (DNT IVB) for fit-for-purpose applications. One crucial issue for OECD application of methods is validation, which for new approach methods (NAMs) requires novel approaches. Here, mechanistic information previously identified *in vivo*, as well as reported neurodevelopmental adversities in response to disturbances on the cellular and tissue level, are of central importance. In this study, we scientifically validate the Neurosphere Assay, which is based on human primary neural progenitor cells (hNPCs) and an integral part of the DNT IVB. It assesses neurodevelopmental key events (KEs) like NPC proliferation (NPC1ab), radial glia cell migration (NPC2a), neuronal differentiation (NPC3), neurite outgrowth (NPC4), oligodendrocyte differentiation (NPC5), and thyroid hormone-dependent oligodendrocyte maturation (NPC6). In addition, we extend our work from the hNPCs to human induced pluripotent stem cell-derived NPCs (hiNPCs) for the NPC proliferation (iNPC1ab) and radial glia assays (iNPC2a). The validation process we report for the endpoints studied with the Neurosphere Assays is based on 1) describing the relevance of the respective endpoints for brain development, 2) the confirmation of the cell type-specific morphologies observed *in vitro*, 3) expressions of cell type-specific markers consistent with those morphologies, 4) appropriate anticipated responses to physiological pertinent signaling stimuli and 5) alterations in specific *in vitro* endpoints upon challenges with confirmed DNT compounds. With these strong mechanistic underpinnings, we posit that the Neurosphere Assay as an integral part of the DNT *in vitro* screening battery is well poised for DNT evaluation for regulatory purposes.

## 1 Introduction

During the last years, enormous scientific and regulatory efforts have been focusing on the establishment of a novel procedure for developmental neurotoxicity (DNT) evaluation (Sachana et al., 2019). The two main drivers for these efforts were the extremely high costs that current DNT guideline studies demand and the resulting overall lack of data, including mechanistic information, that exists for chemicals concerning their influence on brain development. In addition, unique features of the human brain and its development (Rice and Barone Jr., 2000; Dehay and Kennedy, 2007, 2009; Somel et al., 2011; Florio and Huttner, 2014; Pollen et al., 2015; Borrell, 2019) strongly support the general endeavor to use human-derived models for risk decisions in 21st-century toxicity evaluation (National Research Council, 2007). There is a vast amount of data on different compound classes including metals, pesticides, and drugs linking compound exposure to adverse neurodevelopmental outcomes in children, like a drop in IQ or memory and attention deficits (Vorhees et al., 2018). Nevertheless, so far only 110–140 chemicals have been evaluated using *in vivo* DNT guideline studies (Makris et al., 2009; Paparella et al., 2020), while for the majority of the human exposome this data is lacking (Sachana et al., 2021a). Moreover, the contribution of chemical exposure to human neurodevelopmental diseases like autism spectrum or attention deficit hyperactivity disorder has so far only been heavily discussed on an associative basis but not finally mechanistically substantiated (Grandjean and Landrigan, 2006; Abbasi, 2016; Bennett et al., 2016; Gould et al., 2018; Moosa et al., 2018; Cheroni et al., 2020; Masini et al., 2020). Considering the social, societal and economic consequences that DNT entails (Bellinger, 2012; Grandjean and Landrigan, 2014), it is obvious that strategies are needed that allow a faster, more cost-efficient and human-relevant assessment of DNT for closing this obvious data gap.

Efforts for the implementation of DNT *in vitro* alternative methods for hazard identification and risk assessment have been evolving over more than 15 years (Coecke et al., 2007; Lein et al., 2007; Crofton et al., 2011; Bal-Price et al., 2012; Bal-Price et al., 2015a; Fritsche, 2017; Fritsche et al., 2017). According to the proposed paradigm shift in DNT testing (Sachana et al., 2019), a DNT *in vitro* battery (IVB) was assembled based on neurodevelopmental key events (KE; Fritsche et al., 2018b) and assay readiness (Bal-Price et al., 2018). DNT test methods have been assembled (Harrill et al., 2018; Masjosthusmann et al., 2020) and are the basis for a currently prepared guidance document of the Organisation for Economic Co-operation and Development (OECD) with the purpose to shape a framework facilitating the regulatory use of DNT *in vitro* data with fit-for-purpose applications (Crofton and Mundy, 2021). The guidance document rests on two pillars, i.e. 1) the data generated through the experimental work (Masjosthusmann et al., 2020) and 2) the development of a variety of case studies including integrated approaches to testing and assessment (IATA) for screening and prioritization. The OECD guidance document is planned to be published in the first quarter of 2022.

One crucial issue for OECD application of methods is validation (Coecke et al., 2007; Gourmelon and Delrue, 2016; Griesinger et al., 2016). While the traditional practice for assay validation is lengthy and relies on animal data, new approach methods (NAMs) need novel validation approaches. Here, mechanistic information previously identified *in vivo*, as well as reported neurodevelopmental adversities in response to disturbances on the cellular and tissue level, are of central importance (Hartung et al., 2013; Leist and Hartung, 2013). Here the scientific basis of a test method provides the mechanistic rationale for the predictive capacity of the assay. In addition, reliability, defined by the quality of the test method, is a crucial parameter. The scientifically sound, reliable test method also has to be fit-for-purpose implying that the regulatory question is known (Leist et al., 2010, 2014). Lab-to-lab transferability of assays has always been one crucial aspect of assay reliability. However, when e.g. certain robotics equipment is available only in one place, ring trials cannot be performed (Judson et al., 2013).

**FIGURE 1:**
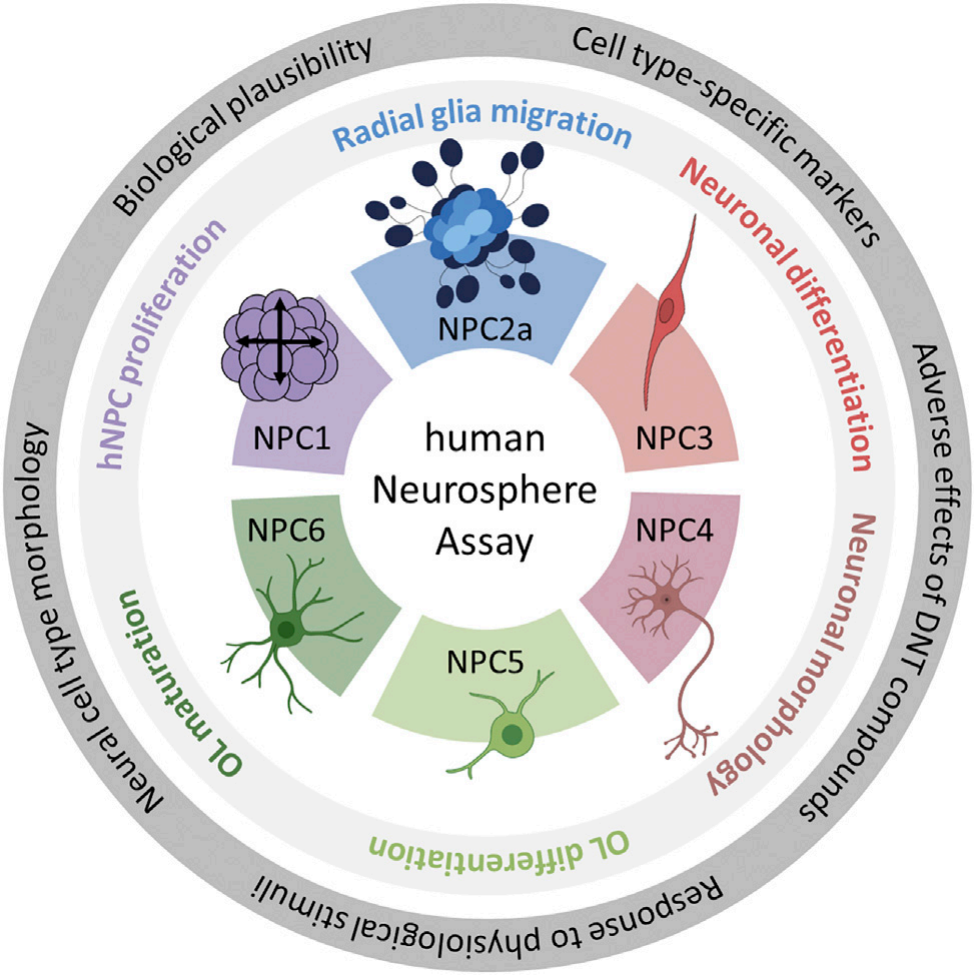
Schematic description of the human Neurosphere Assay test methods and the identification of their scientific bases. The outer ring highlights the five questions which were the basis of the scientific validation of all six assays. The inner ring contains the neurodevelopmental key events modelled in the individual NPC assays. Each color represents one NPC assay. The color scheme is kept throughout the manuscript. Abbreviation: OL, oligodendrocytes. Created with biorender.com.

In this study, we validate the Neurosphere Assay, a high content assay for DNT evaluation, which is part of the DNT IVB (Masjosthusmann et al., 2020), using a mechanistic rationale approach. The Neurosphere Assay is based on human fetal neural progenitor cells (hNPCs) which are cultivated as proliferative neurospheres that have the potential to differentiate into brain effector cells including neurons, astrocytes and oligodendrocytes. Six early human fetal neurodevelopment key events (KEs) which are associated with DNT, are assessed in assays termed NPC1-6. Human NPC proliferation (NPC1ab) is a prerequisite for brain formation, with disturbances causing alterations in brain morphology and microcephaly (de Groot et al., 2005). Radial glia cell migration (NPC2a) generates a scaffold for migrating neurons during the course of corticogenesis and ensures normal brain structure and function. Alterations in this KE cause developmental brain disorders such as heterotopia and lissencephaly (Barkovich et al., 2005). Neuronal differentiation (NPC3) and neurite outgrowth (NPC4) are key cellular features associated with the functional maturation of the CNS. Disturbances in both directions (promotion or inhibition) are considered as adverse and are associated with depressive mood disorders and intellectual disabilities (Song and Wang, 2011; Guidi et al., 2018). Oligodendrocyte differentiation (NPC5) and thyroid hormone-dependent oligodendrocyte maturation (NPC6) are indispensable for the insulation of neuronal axons with disturbances causing demyelination diseases like leukomalacia that severely affect neuronal functioning (Baumann and Pham-Dinh, 2001; Volpe et al., 2011). Besides these DNT-relevant neurodevelopmental KEs, the Neurosphere Assay assesses the mitochondrial function and detects cytotoxicity upon chemical exposure to discriminate specific from unspecific effects (Figure 1).

All individual test method evaluations are automated, and concerning the experimental procedures, i.e. most pipetting steps are performed by a liquid handling system. The NPC2-5 assays are multiplexed. Quantification of differentiated neurons and oligodendrocytes is achieved by automated image analyses of immunostained cells that migrate out of the spheres in 96-well plates using convolutional neuronal networks (CNN; Förster et al., 2021). All endpoints are then analyzed using the Omnisphero software (Schmuck et al., 2017). This automated endpoint evaluation system allows an investigator bias-free, objective and low hands-on-effort identification of specific brain cells that used to be only possible by manual counting. In addition to cell identification, sphere-related endpoints like radial migration can be assessed. We further add data on the novel human induced pluripotent stem cell (hiPSC)-derived NPC (hiNPC) assays (hiNPC1/2) investigating similar endpoints. This hiPSC-based test system provides an unlimited cell source that is thoroughly characterized in a quality-controlled banking process (Tigges et al., 2021) that contributes to increasing the reproducibility of the test results. Furthermore, since iPSCs are reprogrammed from somatic cells (e.g. fibroblasts), the cell source raises fewer ethical concerns regarding its derivation process than primary cell material. However, hiNPCs represent a more immature developmental stage than fetal NPCs, hence they also have distinct applicability domains concerning neurodevelopmental timing.

Here we present the scientific basis for the individual NPC test methods. That the endpoints tested with the Neurosphere Assay are biologically indispensable for normal brain development (biological plausibility) was described in detail earlier (Fritsche et al., 2018b). Therefore, we now focus on the morphology of the different neural cell types, the expression of cell type-specific markers, the responses of the neurodevelopmental processes to physiological stimuli by using signaling pathway modulators and the predictive power to identify adverse effects of known DNT compounds. Together with the biological relevance of the endpoint, these five aspects build the scientific basis of the Neurosphere Assay.

## 2 Materials and Methods

### 2.1 Reagents

Test compounds applied for the validation process are summarized in Table 1. Details for each specific assay are described below.

**TABLE 1:** List of chemicals used in the experimental part, including their sources, catalog numbers, stock concentrations, and solvents.

Reagents	Source	Catalog Number	Solvent	Stock
Ascorbic acid	Merck	A92902	H2O	100 mM
Bisindolylmaleimide 1 (Bis-I)	Merck	203290	DMSO	20 mM
Cadmium chloride	Toxcast library	DTXSID6020226	DMSO	20 mM
N-[N-(3,5-Difluorophenacetyl)-Lalanyl]- S-phenylglycine t-butyl ester (DAPT)	Merck	D5942	DMSO	40 mM
Deltamethrin	Merck	45423	DMSO	20 mM
Epidermal growth factor (EGF)	Thermo Fisher	PHG0313	DPBS +/+	10 μg/ml
Fibroblast growth factor (FGF) basic	R&D Systems	233-FB	0.1% BSA, 1 mM DTT in DPBS +/+	10 μg/ml
Methylmercury(II) chloride	Merck	33368	H2O	20 mM
Narciclasine	Cayman Chemicals	20361	DMSO	20 mM
NH-3	Nguyen et al. (2002)	-	DMSO	1 mM
	Singh et al. (2016)			
PD153035	Merck	SML0564	DMSO	5 mM
PP2	Merck	P0042	DMSO	10 mM
Rotenone	Santa Cruz Biotechnology	203242	DMSO	100 mM
L-3,3′,5 triiodothyronine (T3)	Merck	T2877	1:1 (v/v) 96% EtOH : 1 M HCl	0.3 mM
Tetrabromobisphenol A (TBBPA)	Merck	330396	DMSO	50 mM

### 2.2 Basic hNPC and hiNPC Cell Culture

Primary human NPCs (hNPCs) were isolated from cortices of gestational week 16–19 fetuses and purchased from Lonza Verviers SPRL, Belgium (#PT-2599). The hiPSCs were purchased from Alstem (iPS11) and WiCell (IMR-90, Clone-4). The neural induction of hiPSCs into human-induced neural progenitor cells (hiNPCs) was performed in our laboratory as described in detail in Nimtz et al. (2020). The hNPCs and hiNPCs were cultured as free-floating neurospheres in proliferation medium consisting of DMEM (#31966-021, Thermo Fisher, United States) and Hams F12 (#31765-027, Thermo Fisher, United States) in a 2:1 ratio (v:v) supplemented with 2% B27 (#17504044, Thermo Fisher, United States), 20 ng/ml EGF (#PHG0313, Thermo Fisher, United States), 20 ng/ml FGF basic (#233-FB, R&D Systems, United States), and 100 U/ml penicillin and 100 µg/ml streptomycin (#P06-07100, Pan-Biotech, Germany). Neurospheres were cultured under standard cell culture conditions at 37°C and 5% CO_2_ in 10 cm diameter cell culture dishes coated with poly-2-hydroxyethyl methacrylate (poly-Hema; #P3932, Merck, United States). For cell passaging, once per week, neurospheres were mechanically dissociated into cubicles of 0.2 mm edge length using a McIlwain tissue chopper (#TC752, Campden Instruments, United Kingdom). Neurospheres were supplied with fresh culture medium three times per week, by replacing half of the culture medium.

### 2.3 The Neurosphere Assay (NPC1-6)

#### 2.3.1 Proliferation (NPC1ab)

hNPC and hiNPC proliferation (NPC1ab assay) was scientifically validated by assessing the incorporation of bromodeoxyuridine (BrdU, NPC1b, #11669915001, Roche, Switzerland) into the DNA and by measuring the increase in sphere size (NPC1a; 0, 24, 48, and 72 h) using the Cellomics ArrayScan and the provided HCS Studio Cellomics software (version 6.6.0; Thermo Fisher Scientific). In brief, proliferating NPCs of 300 μm diameter were cultivated for 3 days in 100 µl proliferation medium containing EGF and FGF basic (detailed composition described in the basic cell culture section). One NPC neurosphere was cultivated in 100 µl medium in a well of a poly-Hema-coated 96-well plate and 4–5 technical replicates were prepared for each experimental condition. Proliferating NPCs were further exposed to the EGF receptor inhibitor PD153035 (0.01, 0.16 and 0.64 µM), known DNT-positive chemicals with known modes of action, i.e. cadmium chloride (0.03–20 µM) and rotenone (0.01–2.22 µM), or the respective solvent (solvent control) over the whole 3 days. As an endpoint-specific positive control, confirming that the assay detects reductions in NPC proliferation, NPCs were cultivated in medium without growth factors (w/o growth factors). For the assessment of the sphere size, images of neurospheres were taken daily using an inverted microscope CKX41 (Olympus) with a ×100 magnification. Detailed descriptions of the NPC1ab and iNPC1ab assays can be found in the DNT *in vitro* testing battery report (Masjosthusmann et al., 2020) and in (Hofrichter et al., 2017), respectively. Besides proliferation, cell viability (CellTiter-Blue Assay (CTB), #G8081, Promega, Madison, United States) and cytotoxicity (CytoTox-ONE Homogeneous Membrane Integrity Assay; #G7891, Promega, Madison, United States) were assessed simultaneously.

Flow cytometry analyses were performed to confirm the cell type-specific marker expression in proliferating hNPC and hiNPC neurospheres. Neurospheres (hNPCs in passage 4, hiNPCs in passage 5) were singularized with accutase (Stemcell Technologies, Canada) for 20 min at 37°C on an orbital shaker (800 rpm) and stained with viability stain 510 (#564406, BD Bioscience, Germany), anti-Nestin-Alexa647 antibody (#560341, BD Bioscience, Germany) and anti-Sox2-PerCP-Cy5.5 antibody (#561506, BD Bioscience, Germany). The antibodies were all diluted 1:20 in BD Pharmingen stain buffer (BD Bioscience, Germany) except for the viability stain (1:1000 in DPBS −/−, #14190144, Thermo Fisher, United States). Samples were analyzed using a BD FACSCanto II (BD Bioscience, Germany) and FlowJo software (10.8.0). Debris, doublets, and dead cells were discarded during the gating process. 20000 cells were analyzed per sample.

#### 2.3.2 hNPC Differentiation and Immunocytochemical Staining (NPC2-5)

For the initiation of cell differentiation into neurons, oligodendrocytes, and astrocytes (Moors et al., 2009; Breier et al., 2010), 0.3 mm hNPC neurospheres were transferred into 96-well plates coated with 0.1 mg/ml poly-D-lysine (#P0899-50MG, Merck, United States) and 12.5 µg/ml laminin (#L2020-1MG, Merck, United States). The following differentiation medium was prepared and used for neurosphere cultivation: DMEM (#31966-021, Thermo Fisher, United States) and Ham’s F12 (#31765-027, Thermo Fisher, United States) in a 2:1 ratio (v:v) supplemented with 1% N2 (#17502-048, Thermo Fisher, United States) and 100U/mL penicillin and 100 µg/ml streptomycin (#P06-07100, Pan-Biotech, Germany). After 5 days of differentiation, migrated cells were fixed with 4% paraformaldehyde for 30 min at 37°C and stained with antibodies against β(III)tubulin (neurons) and O4 (oligodendrocytes) as previously described in detail by (Klose et al., 2021b; 2021a). In brief, unspecific binding sides on the fixated cells were blocked with 10% goat serum (GS, #G9023-10ml, Merck, United States) in PBS for 30 min at 37°C. Primary antibodies against β(III)tubulin (1:400, rabbit anti-β(III)tubulin monoclonal antibody [EP1569Y]-Alexa Fluor^®^ 647, #ab190575, Abcam, United Kingdom) and O4 (1:400, mouse anti-O4 IgM, #MAB1326, R&D systems, United States) were incubated overnight in PBS containing 0.01% Triton-X and 2% GS at 4°C. After three washing steps with PBS, the cells were incubated with the secondary antibody for the O4-staining (1:400, goat anti-mouse IgM-Alexa Fluor^®^ 488, #A-21042, Thermo Fisher, United States) and Hoechst33258 (1:100, #94403-1ML, Merck, United States) in PBS containing 2% GS for 60 min at 37°C. For the staining of radial glia, fixated cells were blocked with 10% GS in PBS for 30 min at 37°C and stained with antibodies against nestin (1:200, Alexa Fluor^®^ 647 mouse anti-nestin, #560393, BD Biosciences, United States), Ki67 (1:500, Ki67 (8D5) mouse mAb, #9449, Cell Signaling Technologies, United States) or GFAP (1:200, anti-GFAP mouse (GA5) antibody, #G9269, Merck, United States) overnight in PBS containing 0.01% Triton-X and 2% GS at 4°C. After three washing steps with PBS, the cells were incubated with the secondary antibodies for Ki67 (1:400, goat anti-mouse IgG 488, #A-11001, Thermo Fisher, United States) and GFAP (1:400, goat anti-rabbit IgG 488, #A-11008, Thermo Fisher, United States) and Hoechst33258 (1:100, #94403-1ML, Merck, United States) in PBS containing 2% GS for 60 min at 37°C. All pictures of immunocytochemical stainings were acquired with the High Content Analysis (HCA) platform Cellomics ArrayScan using a 200-fold magnification, a resolution of 552×552 pixel and the provided HCS Studio Cellomics software (version 6.6.0; Thermo Fisher Scientific).

#### 2.3.3 hiNPC Differentiation and Migration (hiNPC2a+3)

Differentiation of hiNPCs was performed as described above for primary hNPCs, with the exception that hiNPCs were cultivated in CINDA medium containing DMEM (#31966-021, Thermo Fisher, United States) and Ham’s F12 (#31765-027, Thermo Fisher, United States) in a 2:1 ratio (v:v) supplemented with 1% N2 (#17502-048, Thermo Fisher, United States), 2% B27 (#17504044, Thermo Fisher, United States), 100 U/ml penicillin and 100 µg/ml streptomycin (#P06-07100, Pan-Biotech, Germany), 5 mM creatine monohydrate (#C3630, Merck, United States), 100 U/mL Interferon-γ (#300-02, PeproTech, Germany), 20 ng/ml neurotrophin-3 (#450-03, PeproTech, Germany), 300 µM dibutyryl-cAMP (#D0260, Merck, United States) and 20 µM ascorbic acid (#A5960, Merck, United States). The neural induction of human induced pluripotent stem cells (hiPSCs) into human induced neural progenitor cells (hiNPCs) is described in detail in Bartmann et al. (2021) and Nimtz et al. (2020). After 3 days of differentiation, cells were fixed with 4% paraformaldehyde for 30 min at 37°C, washed four times with PBS, and stained with S100β antibody (1:500, rabbit anti-S100 beta antibody [EP1576Y], #ab52642, Abcam, United Kingdom) in 0.05% PBS-T with 3% GS overnight at 4°C, followed by five PBS washing steps, before incubation with the secondary antibody (1:500, goat anti-rabbit IgG 488, #A-11008, Thermo Fisher, United States) in PBS with 2% GS and 1% Hoechst 33258 (1:100, #94403-1ML, Merck, United States) for 60 min at room temperature. After 5 washing steps with PBS, cells were stained with the conjugated β(III)tubulin antibody (1:400, rabbit anti-β(III)tubulin monoclonal antibody [EP1569Y]-Alexa Fluor^®^ 647, #ab190575, Abcam, United Kingdom) in PBS with 2% GS. After 5 washing steps with PBS, images of immunocytochemical stainings were acquired as described for primary hNPCs.

For the scientific validation of the hiNPC migration assay, hiNPCs were exposed to either EGF (0.5–1 ng/ml) alone or in combination with the EGFR-inhibitor PD153035 (1–2 µM), the SRC- kinase inhibitor PP2 (10 µM), narciclasine (0.0001–0.1 µM) or the respective solvent (solvent control). The migration distance was assessed after 24, 48 or 72 h as described for hNPC migration below.

#### 2.3.4 hNPC Migration (NPC2)

Upon plating of hNPC neurospheres on PDL-laminin matrices, NPCs radially migrate out of the sphere core, thereby adapting a radial glia-like morphology and forming a circular migration area. The migration distance of radial glia cells (RG, NPC2a) is assessed manually after 72 h using bright-field microscopy and automated after 120 h by analyzing the ICC stainings with the software Omnisphero as previously described by Schmuck et al. (2017). In brief, RG migration is assessed manually on bright-field pictures, taken with the Cellomics ArrayScan using a 50-fold magnification, by measuring the radial distance of the furthest migrated cells to the sphere core as number of pixels following conversion into µm using Fiji Image J software (Schneider et al., 2012). After 120 h, RG migration is evaluated automatically by defining the area of Hoechst33258-stained nuclei as the migration area of this particular sphere using the Omnisphero software. Additionally, the migratory capacity of neurons (NPC2b) and oligodendrocytes (NPC2c), defined as the mean distance of all neurons/oligodendrocytes within the migration area divided by the RG migration distance, is automatically assessed after 120 h. The validation of the NPC2b and NPC2c assay is not included in this manuscript.

For the scientific validation of the NPC2a assay, hNPCs were exposed to human-relevant pathway modulators as well as known DNT-positives during the 5 days of differentiation. Neurospheres were differentiated in presence of 1) epidermal growth factor (EGF, 0.5–1 ng/ml) alone or in combination with the EGF receptor-inhibitor PD153035 (1–2 µM), 2) the Src-kinase inhibitor PP2 (10 µM), 3) increasing concentrations of methylmercury (MeHg, 0.003–2.22 µM) or 4) the respective solvent (solvent control). Besides RG migration analysis, cytotoxicity was assessed.

#### 2.3.5 Neuronal and Oligodendrocyte Differentiation and Neuronal Morphology (NPC3-5)

Multiplexed with the assessment of RG migration after 120 h (NPC2a), further endpoints can be assessed in an automated way using different software tools. The endpoints NPC3-5 model neuronal differentiation (NPC3) and morphology (NPC4), as well as oligodendrocyte differentiation (NPC5) after 120 h of differentiation.

After staining of the differentiated cells with the above-mentioned antibodies and subsequent image acquisition with the Cellomics ArrayScan (see section “*hNPC Differentiation and Immunocytochemical staining*”), a series of separate images were edited together to create one image per well, including all three channels (nuclei (Hoechst33258), neurons (Alexa647^®^), oligodendrocytes (Alexa488^®^)). For this step, the high-content analysis (HCA) tool Omnisphero was used (Schmuck et al., 2017). Based on the cells with Hoechst-positive nuclei migrating out of the sphere core and the formed circular migration area, RG migration was calculated for each sphere after 120 h. Neuronal (NPC3) and oligodendrocyte (NPC5) differentiation is defined by the number of cells stained for β(III)tubulin and O4, respectively, as a percentage of the total nuclei count within the migration area. The stained neurons and oligodendrocytes are identified using two convolutional neural networks (CNN) based on the Keras architecture implemented in Python 3, which were trained by historical handpicked data (Förster et al., 2021). The number of nuclei was determined using the SpotDetector (V4.1) bio-application of the HCS Studio Cellomics software (version 6.6.0, Thermo Fisher Scientific). All neurons identified by the CNN were additionally analyzed regarding their morphology by assessing their neurite length and area (NPC4).

For the scientific validation of the NPC3 assay, neurospheres were differentiated in presence of DAPT (0.01 µM–10 µM), narciclasine (0.00014 µM–0.1 µM), or the respective solvent (solvent control). For the scientific validation of the NPC4 assay, neurospheres were differentiated in presence of narciclasine (0.00014 µM–0.1 µM), bisindolylmaleimide 1 (0.027–20 µM) or the respective solvent (solvent control). For the scientific validation of the NPC5 assay, neurospheres were differentiated in presence of DAPT (0.01 µM–10 µM), 100 µM ascorbic acid, deltamethrin (0.027–20 µM), tetrabromobisphenol A (0.027–20 µM) or the respective solvent (solvent control).

#### 2.3.6 Oligodendrocyte Maturation Assay (NPC6)

The methodology is described in detail in (Dach et al., 2017; Klose et al., 2021b). In brief, hNPCs were plated on 8-chamber slides (five spheres per chamber) and 24-well plates (10 spheres per well) in differentiation medium containing either solvent or 3 nM triiodothyronine (T3) and incubated for 5 days on PDL-laminin matrices. To test for thyroid hormone disruption, hNPCs were additionally differentiated in presence of T3 with or without increasing concentrations of the thyroid hormone receptor antagonist NH-3 (4–400 nM) or the flame retardant TBBPA (0.01–1 µM). After 5 days, immunocytochemical stainings for oligodendrocytes (O4) and cell nuclei (Hoechst33258) were performed in the 8-chamber slides as described above. Imaging of stained 8-chamber slides was performed using the Cellomics ArrayScan VTI instrument (Thermo Fisher Scientific) and the software Omnisphero (Schmuck et al., 2017). For two defined areas (1098 mm x 823 mm size) within the migration area, the oligodendrocyte number was calculated and expressed as a percentage of the total number of nuclei. Oligodendrocyte percentages were averaged per sphere and the mean and standard deviation were calculated for the five spheres per chamber.

From the spheres plated within the 24-well plate, total RNA was extracted and 150 ng were transcribed into cDNA using the RNeasy Mini Kit (#74106, Qiagen, Germany) and the Quantitect Reverse Transcription Kit (#205313, Qiagen, Germany) according to the manufacturer’s instructions. Quantitative real-time polymerase chain reactions (qRT-PCR) were performed with the QuantiFast SYBR Green PCR Kit (#204054, Qiagen, Germany) and the Rotor-Gene Q Cycler (Qiagen, Germany) using primers for ACTB (fw: CAG​GAA​GTC​CCT​TGC​CAT​CC, rev: ACC​AAA​AGC​CTT​CAT​ACA​TCT​CA), MBP (fw: CAG​AGC​GTC​CGA​CTA​TAA​ATC​G, rev: GGT​GGG​TTT​TCA​GCG​TCT​A). Gene expression was quantified with the copy number method and MBP expression was normalized to 10.000 ACTB copy numbers (Dach et al., 2017; Klose et al., 2021b).

The maturation quotient (Q_M_) is then calculated as MPB copy numbers divided by the percentage of oligodendrocytes within the hNPC differentiated culture. Therefore, an increase in the Q_M_ represents an increase in oligodendrocyte maturation.

#### 2.3.7 Mitochondrial Activity and Cytotoxicity Assays

Mitochondrial activity and cytotoxicity were assessed in parallel to the specific endpoints of the Neurosphere Assay to discriminate specific compound effects from unspecific effects originating from necrosis or reduced cell viability. After the respective days of chemical exposure, mitochondrial activity was assessed using the Alamar blue assay (CellTiter-Blue Assay (CTB), #G8081, Promega, United States). In parallel, cytotoxicity was determined by measuring the release of lactate dehydrogenase (LDH) from cells with damaged membranes (CytoTox-ONE Homogeneous Membrane Integrity Assay; #G7891, Promega, United States). As lysis control for the LDH assay, neurospheres were incubated for 45 min with 0.2% Triton X-100. Fluorescence was measured with a Tecan infinite M200 Pro reader (ex: 540 nm; em: 590 nm). The relative fluorescence unit (RFU) values of the replicates were averaged and medium without cells was used to correct for background fluorescence. Of note, impaired radial glia migration and reduced nuclei count correlate with a reduced CTB value as a consequence of the diminished cell number within the migration area (Fritsche et al., 2018a; Klose et al., 2021a). Therefore, in the case of a compound inhibiting radial glia migration or reducing the nuclei count, the CTB assay is an inadequate measure of viability and thus the LDH assay alone should be used as the reference to identify DNT-specific effects (Klose et al., 2021a). In the figures, except for Figures 5F+G and Figure 7F, only cytotoxicity is displayed.

#### 2.3.8 Statistics

For all hNPC experiments, at least two different individuals (hNPC donors) were used and for all hNPC and hiNPC experiments, at least three independent biological replicates with at least three technical replicates each were performed. Experiments were defined as independent if they were generated with hNPCs from different individuals or a different passage number. Results are presented as mean ± SEM unless otherwise indicated. For calculating dose-response curves, a sigmoidal curve fit was applied using GraphPadPrism software. Statistical significance was calculated using one-way ANOVA with Bonferroni’s post hoc tests or two-tailed Student’s t-tests (*p* ≤ 0.05 was termed significant).

## 3 Results and Discussion

In the next paragraphs, we will guide through the endpoints of the Neurosphere Assays, starting with the human NPC assays that are based on primary human fetal NPCs. Succeeding, we present endpoints of a novel iNeurosphere Assay, which is based on human induced pluripotent stem cells. During method development, we proceeded according to the Guidance Document on Good *In Vitro* Method Practices (GIVIMP) principles, to ensure predictivity and reproducibility of the test methods (OECD, 2018; Pamies et al., 2022). The description of each Neurosphere Assay endpoint follows the same rationale. First, the relevance of the respective endpoint for brain development is described. Second, *in vitro* morphologies and expressions of respective markers corresponding to the individual cell types and test methods are shown. Third, endpoint responses to a selection of physiologically pertinent signaling stimuli during neurodevelopmental processes are demonstrated. These data underscore the biological relevance of the individual endpoints. Fourth, examples of adverse effects of DNT compounds on neurosphere endpoints are displayed. These data are important building blocks for scientific validation of DNT test methods since they contribute to the scientific basis and applicability domains of the studied neurodevelopmental processes and hence increase confidence in their usage.

### 3.1 NPC Proliferation (NPC1)

Proliferation is one of the essential neurodevelopmental processes during brain development and comprises the increase in cell number through cell growth and division (Homem et al., 2015). Disturbances in both directions (decrease and increase of proliferation) may result in neurodevelopmental disorders such as microcephaly or megalencephaly, respectively. Microcephaly is manifested by a severe reduction in brain size and was linked to prenatal exposure of human fetuses to the Zika virus (Devakumar et al., 2018). Megalencephaly, on the other hand, is defined as increased growth of cerebral structures during development and is associated with metabolic disorders such as L-2-hydroxyglutaric aciduria (Pavone et al., 2017). Both microcephaly and megalencephaly may result in severe neurological disabilities such as global developmental delay, seizures, deficits in language development and social interactions (Guerrini and Dobyns, 2014).

**FIGURE 2:**
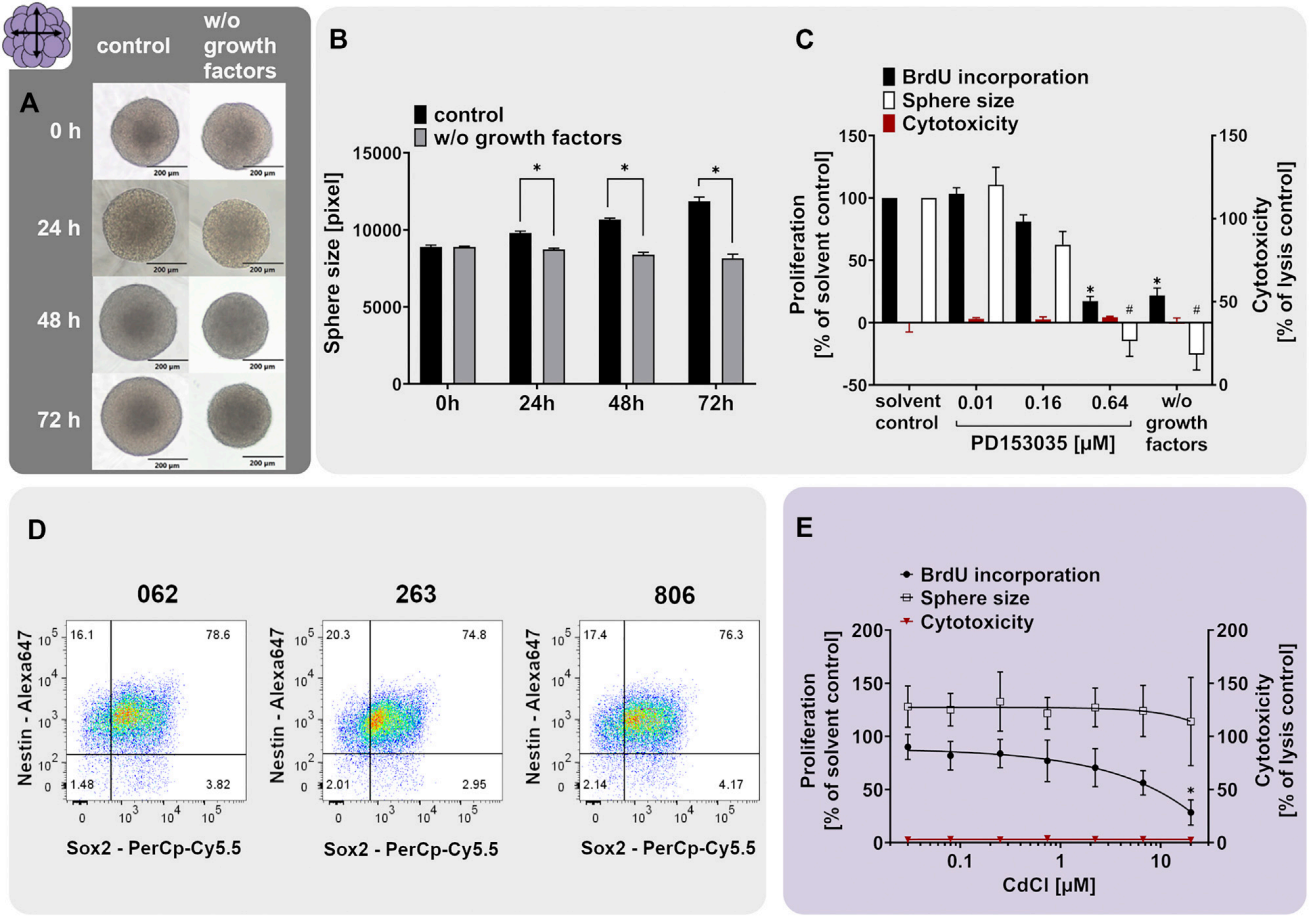
The NPC1ab assay identifies chemicals disturbing NPC proliferation. **(A+B)** Primary hNPC neurospheres (Lonza, Verviers, Belgium) were cultivated for 3 days in proliferation medium containing 20 ng/ml of the growth factors EGF and FGF (control) or in medium without growth factors (w/o growth factors). Representative pictures **(A)** and quantifications of the sphere size **(B)**, as assessed within the NPC1a assay, showed that growth factors are necessary for hNPC proliferation. **(D)** Proliferating hNPC neurospheres issued from three different individuals (062, 263, and 806) were analyzed using flow cytometry analysis, confirming high expression of the neural stem/progenitor markers nestin and SOX2. The percentage of double-positive cells is indicated in the upper right quartile. **(C, E)**. Exposure of proliferating hNPCs for 3 days to increasing concentrations of **(C)** the EGFR inhibitor PD153035 (0.01–0.64 µM) or **(E)** cadmium chloride (0.027–20 µM) concentration-dependently decreased hNPC proliferation compared to the respective solvent controls (adapted from Masjosthusmann et al., 2020). Proliferation was assessed by sphere size increase (NPC1a) and BrdU incorporation into the DNA (NPC1b). The values of the chemical-treated conditions are expressed as % of the respective solvent controls. Cytotoxicity (LDH release) was assessed in parallel and is depicted as % of a lysis control (spheres treated with 0.2% Triton-X100). Data are presented as mean ± SEM. Statistical significance was calculated using one-way ANOVA **(C, E)** and two-tailed Student’s t-tests **(B)**. A *p*-value below 0.05 was termed significant. * and ^#^ significantly changed compared to the solvent control of the respective endpoint if not marked otherwise.

Neurospheres are valuable 3D test systems to study NPC proliferation since they are highly proliferative in suspension culture in the presence of growth factors (Reynolds et al., 1992). For our specific neurosphere test system (Lonza, Verviers, Belgium), expression of the cell type-specific CNS neural stem and progenitor cell markers nestin and SRY-box 2 (SOX2) in proliferating hNPCs was confirmed (Figure 2D). Nestin is an intermediate filament protein type IV (Lendahl et al., 1990) used as a molecular marker for neuroepithelial stem cells and CNS progenitors. When human multipotent CNS progenitors differentiate into neurons and glial cells, nestin expression is rapidly downregulated *in vivo* (Dahlstrand et al., 1995) confirming its usefulness as a neural progenitor cell marker. SOX proteins comprise a group of transcription factors conserved throughout evolution. SOX2 is a marker for proliferating CNS progenitors and its overexpression inhibits neuronal differentiation (Pevny and Placzek, 2005). The primary hNPCs used in this study were double-positive for nestin and SOX2 as shown in Figure 2D. In total, 76.6, 74.8, and 76.3% of cells issued from the three different individuals, respectively, were double-positive for the two markers, and only 1.48, 2.01, and 2.14% of cells expressed none of them, hence confirming their resemblance to neural progenitors *in vivo*. The expression of nestin and SOX2 was assessed in primary hNPCs previously (Hofrichter et al., 2017). Although the percentage of cells expressing neither of the two markers was comparable in these two studies, the average percentage of double-positive cells was lower in the present study (75.9% in the present study versus 96.3% (Hofrichter et al., 2017)). This might be explained by a higher passage number (4) of hNPCs used in this study (i.e. the highest passage usually used within the Neurosphere Assay) in comparison with passage 0 used in the study of (Hofrichter et al., 2017). In addition to their expected marker expression, hNPCs exert the expected morphology (Figure 2A). Neurospheres of a few hundred µm in diameter consist of individual cells (e.g. one neurosphere with 300 µm in diameter contains 2.6 x 10^3^ cells; (Moors et al., 2009)) and display a perfectly round shape with no disintegrated borders.

The proliferative capacity of hNPCs was assessed by cultivating them in either medium supplemented with (control) or deprived of (w/o growth factors) the human growth factors EGF and FGF basic (20 ng/ml each). Human NPCs cultivated for 3 days in control medium increased their size on average by 33%, whereas hNPCs cultivated in growth factor-deprived medium (w/o growth factors) did not proliferate and even slightly shrunk in size by 8.2% (Figures 2A,B). Direct measurements of proliferation by BrdU incorporation indicated a 78.1% decrease in BrdU incorporation in spheres growing in the absence (w/o growth factors) compared to the presence (solvent control) of growth factors (Figure 2C). The proliferative capacity of Lonza hNPCs was reported previously (Moors et al., 2009; Baumann et al., 2015; Klose et al., 2021a).

The neurodevelopmental process of proliferation is guided by various signaling pathways including the epidermal growth factor receptor (EGFR) signaling (Ayuso-Sacido et al., 2010). To elucidate if EGFR mediates this proliferative cell response in hNPC, we assessed hNPC proliferation in presence of the EGFR inhibitor quinazoline PD153035. PD153035 antagonized the EGF-induced hNPC proliferation, as assessed via BrdU incorporation and sphere size increase, without inducing cytotoxicity. This confirms that EGFR signaling regulates hNPC proliferation *in vitro* (Figure 2C). EGFR signaling generally regulates cell proliferation, growth, differentiation and cell survival (Oda et al., 2005). In the developing brain, the EGFR is increasingly expressed over time (Romano and Bucci, 2020) and is mainly found in proliferating and migratory brain regions (Kornblum et al., 1997; Caric et al., 2001). The EGFR is therefore indispensable for proper rodent brain development (Romano and Bucci, 2020). Specifically, the proliferation of murine neural stem cells and nestin^+^ progenitor cells was previously increased by EGFR signaling *in vitro* (Sun et al., 2005; Ayuso-Sacido et al., 2010)*. In vivo*, EGF induced proliferation of stem cells and progenitors in the murine fourth ventricle and central canal of the spinal cord (Martens et al., 2002) and ependymal precursor cells of the adult rat spinal cord (Kojima and Tator, 2000). Moreover, PD153035 reportedly suppressed proliferation of murine neural stem cells *in vitro* (Tropepe et al., 1999)*.* These data—especially from the *in vivo* studies—support the importance of the EGFR pathway for NPC proliferation.

As a chemical exerting adverse effects on hNPC proliferation, we selected cadmium chloride. Prenatal exposure to cadmium chloride is associated with a lower child intelligence score (Kippler et al., 2012b), memory deficits, and learning disabilities in children (Tian et al., 2009). In rodents, cadmium causes behavioral and neurotoxicological changes (Dési et al., 1998). Hence, it is listed amongst the compounds triggering DNT (Mundy et al., 2015; Aschner et al., 2017). Cadmium is acting via the induction of oxidative stress, thus causing cell death and affecting mTOR, Erk1/2, and JNK signaling pathway activity (Kippler et al., 2012a; Leal et al., 2012). In mouse neural stem/progenitor cells, cadmium remarkably influenced the expression of genes related to cell growth, proliferation, cell cycle, and survival (Deng et al., 2020). In the present study, concentration-dependent inhibition of hNPC proliferation was observed following exposure to cadmium chloride compared to the solvent control without any observed cytotoxicity (Figure 2E adapted from Masjosthusmann et al. (2020)). Of note, the effects on NPC1b (BrdU incorporation) were much more pronounced compared to NPC1a (sphere size), highlighting that NPC1b is the more sensitive endpoint since differences in DNA replication by far precede the microscopic changes. The hNPC proliferation assay previously identified numerous compounds eliciting adverse effects on the proliferation process e.g. MeHgCl, arsenic, methylazoxy methanol acetate, NaAsO_2_ (Baumann et al., 2016), the flame retardants EHDPHP and TCP (Klose et al., 2021a) and a variety of compounds in a large screening study (Masjosthusmann et al., 2020). These studies support the usefulness of the 3D hNPC test system for assessing the effects of compounds on NPC proliferation.

### 3.2 Radial Glia Migration (NPC2a)

Fetal cortex development is characterized by different migratory processes mainly involving radial glia cells (RG) and neurons (Borrell and Götz, 2014; Falk and Götz, 2017). Human RG exhibit two distinct functions, which are prerequisites for cortex development, especially the higher organization of the human brain: 1) due to their self-renewing capacity, RG increase the cortical cell pool before terminally differentiating into neurons and glial cells. This leads to cortical expansion, increases neurogenesis and causes the characteristic folded cerebral cortex architecture in gyrencephalic species like humans. 2) due to their migratory capacity, RG form scaffolds for migrating neurons and hence represent the pillars of cortex formation (Borrell and Götz, 2014; Falk and Götz, 2017). As a consequence of disturbance of RG function during human brain development, neurodevelopmental disorders such as heterotopia and lissencephaly can develop (Barkovich et al., 2005; Matsumoto et al., 2017; Ferent et al., 2020).

**FIGURE 3:**
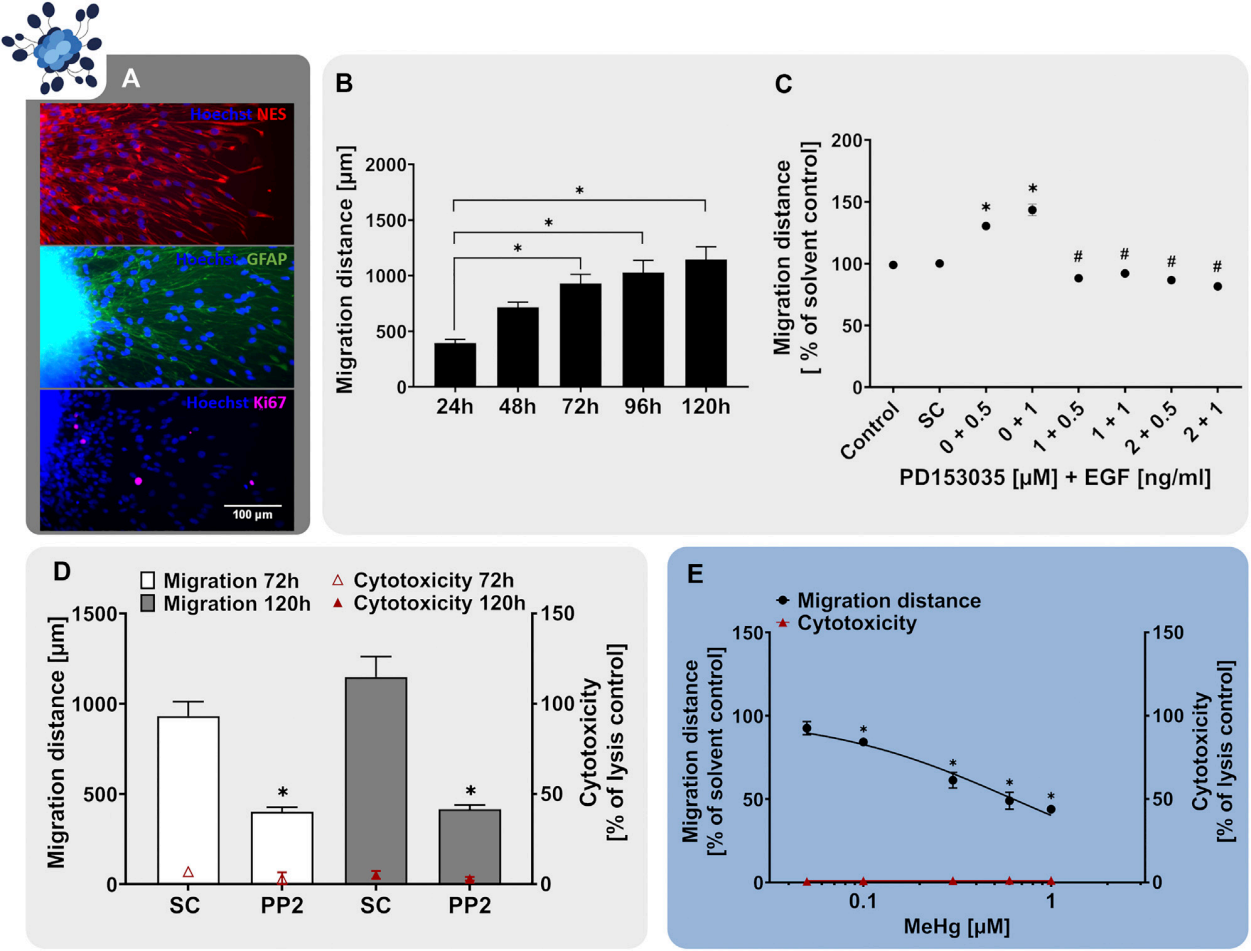
The NPC2a assay identifies chemicals disturbing radial glia migration. Primary hNPCs were differentiated on PDL-laminin-coated 96-well plates in the absence of growth factors. **(A)** After 24 h, immunocytochemical stainings for nestin, GFAP and Ki-67 were performed confirming radial glia-typic marker expression and morphology. Nuclei were counterstained with Hoechst33258. **(B)** Over the 5 days of differentiation, cells radially migrate out of the sphere core and form a circular migration area. The migration distance of hNPCs increased gradually over time, as assessed by determining the distance from the sphere core to the furthest migrated cells at four opposite positions in brightfield images every 24 h **(C)** hNPC migration over 5 days was assessed in presence of EGF (0.5–1 ng/ml) alone, in combination with the EGFR inhibitor PD153035 (1–2 µM), or the respective solvent. While EGF increased hNPC migration compared to the solvent control, PD153035 inhibited the EGF-induced effect. **(D)** A negative effect of the SRC-family kinase inhibitor PP2 on hNPC migration was confirmed by differentiating hNPCs for 3 and 5 days in presence of 10 µM PP2 or the respective solvent (SC). **(E)** hNPCs differentiation in presence of increasing concentrations of methyl-mercury (MeHg, 0.005–1 µM) for 3 days concentration-dependently reduced the migration distance (adapted from Fritsche et al., 2018a). For **(D,E)**, cytotoxicity (LDH release) was assessed in parallel and is depicted as % of a lysis control (differentiated hNPCs treated with 0.2% Triton-X100). Data are presented as mean ± SEM. Statistical significance was calculated using one-way ANOVA **(B, E)** and two-tailed Student’s t-tests **(C, D)**. A *p*-value below 0.05 was termed significant. *significantly changed compared to the respective solvent control. ^#^significantly changed compared to the respective EGF concentration.

Neurospheres are well-suited cell systems for studying neural cell migration since without any additional cues the cells start wandering out of the spheres once they are plated on a suitable matrix (Zhou and Chiang, 1998; Kukekov et al., 1999). In our human neurosphere test system, we established a RG migration test method (NPC2a assay) that specifically measures the migration distance travelled by RG cells (Moors et al., 2007, 2009; Baumann et al., 2015). In our studies, plating of hNPC neurospheres on poly-D-lysine/laminin-coated matrices initiates cell migration in radial trajectories, forming a circular migration area around the sphere core. After 24 h, the migrated cells exhibit the characteristic elongated RG-like morphology and express the RG-markers nestin and GFAP as well as the proliferation marker Ki-67 (Figure 3A). *In vivo*, RG are highly polarized and have a particular elongated morphology since they form processes extending from the apical to the basal side of the cortex (Ferent et al., 2020). In accordance with that, the nestin- and GFAP-positive cells migrating out of the hNPC sphere core display an active growth cone protrusion which diverges from the cell body to explore the vicinal environment (Figure 3A; Baumann et al. (2015)). The migratory potential of the hNPC-derived RG is preserved *in vitro* over the time-course of at least 120 h (Figure 3B). However, a decrease in migration speed can be observed after the first 24 h. Since the specific RG architecture provides a scaffold supporting neuronal migration during cortex development, the correct formation and maintenance of the RG scaffold is crucial for the organization of neuronal networks and disturbances correlate with cortical malformations such as human lissencephaly, polymicrogyria and heterotopia (Ferent et al., 2020). Therefore, RG migration (NPC2) is a fundamental neurodevelopmental key event, which is indispensable in a predictive testing battery identifying chemical-induced DNT.

During human brain development, migratory processes are regulated by various signaling pathways, whose activity should be preserved within a predictive *in vitro* model. Similar to NPC proliferation, migration of neural stem cells is regulated by EGF, exerting its actions through the EGFR (Ayuso-Sacido et al., 2010). The EGFR is expressed not only in proliferating but also in differentiating hNPC and was identified as a human-relevant key regulator in a gene-gene interaction network involved in hNPC migration together with SRC-kinase (Masjosthusmann et al., 2018). Studies on EGFR knockout mice reported a decrease in brain size, supporting the involvement of the EGFR in migratory processes during cortical development (Kornblum et al., 1998). Moreover, intraventricular administration of EGF caused migration of subependymal NPCs from the lateral ventricle into the adjacent neural tissue in the adult mouse brain (Craig et al., 1996). Similarly, exposure to EGF (0.5–1 ng/ml) after neurosphere plating enhanced hNPC migration compared to the solvent control (Figure 3C). In addition, co-administration of the EGFR-inhibitor PD153035 (1–2 µM) antagonized the EGF-induced migratory stimulating effect demonstrating EGF action on NPC migration via the EGFR. As a second human-relevant key regulator of migration, which is expressed in hNPCs (Masjosthusmann et al., 2018), we assessed the effects of SRC-family kinase inhibition on RG migration. SRC-family kinases are fundamental for brain development and disruption of their activity correlates with defects in radial migration and reeler-like malformations of cortical development (Jossin et al., 2003; Kuo, 2005; Wang et al., 2015). Exposure of migrating hNPCs to the SRC-family kinase inhibitor PP2 reduced hNPC migration to 40% of the solvent control without causing any signs of cytotoxicity (Figure 3D; Moors et al., 2007). Our results indicate that human-relevant signaling pathways involved in neurodevelopmental migratory processes *in vivo* (EGFR and SRC) are preserved in the hNPC-derived cells *in vitro* supporting the applicability of the NPC2a assay to study cell migration during development.

In addition to studying signaling pathways, the NPC2a assay is also able to identify chemicals evidently disturbing migratory processes upon chemical exposure. Prenatal exposure to MeHg causes severe neurological symptoms including intellectual disabilities and cerebral palsy in children (Harada, 1978). Investigations of brain autopsy samples confirmed that exposure to MeHg perturbed cell migration and disorganized neocortical layering (Choi et al., 1978), which was verified in animal models (Kakita et al., 2002). Dysplasia and abnormal cortical cytoarchitecture have been attributed to a MeHg-mediated genetic reprogramming of signaling pathways regulating neural development. Hence disrupting the cerebral cortical organization, disturbing migratory processes and causing heterotopia (Choi et al., 1978; Rand et al., 2009; Faustman et al., 2012). One signaling pathway affected by MeHg exposure is the Notch receptor pathway, which controls cell fate decisions, proliferation, migration and neurite outgrowth during neural development (Bland and Rand, 2006). Moreover, MeHg disturbs the cytoskeletal organization involved in cell migration by disrupting the assembly and polymerization of microtubules (Choi, 1991). Exposure of differentiating hNPCs to MeHg resulted in a concentration-dependent reduction of RG migration at *in vivo* relevant concentrations (Figure 3E adapted from Fritsche et al. (2018a), Moors et al. (2009), Baumann et al. (2016)). No cytotoxicity was observed in the tested concentration range, indicating a specific effect of MeHg on hNPC migration. As demonstrated with MeHg, the NPC2a assay is able to detect specific alterations in cell migration and therefore allows for the detection of chemically-induced disruption of migration in the context of brain development. Besides RG migration (NPC2a) the Neurosphere Assay covers the endpoints neuronal (NPC2b) and oligodendrocyte migration (NPC2c). However, for these endpoints, the identification of signaling pathways and model substances is still ongoing, which is the reason why they are not included in this study.

### 3.3 Neuronal Differentiation and Morphology (NPC3+4)

During cortex development, NPCs including RG cells eventually lose their proliferative capacity and terminally differentiate into neural effector cells, i.e. neurons and glia cells (oligodendrocytes and astrocytes). Neurons then migrate alongside the scaffold of RG to their final destinations, to generate the different cortical layers (Rakic, 1972; Gilmore and Herrup, 1997). The generation of sufficient numbers of neurons is a prerequisite for the functionality of neuronal networks and associated learning and memory functions (Berdugo-Vega et al., 2020). Therefore, disturbed neurogenesis manifests in several behavioral disorders such as depression (Song and Wang, 2011) or the intellectual disabilities of patients with Down Syndrome (Guidi et al., 2018; Stagni et al., 2018). In addition, an elevation in neurogenesis is a major driver of epileptogenesis (Jessberger and Parent, 2015). Hence, the correct balance of neurogenesis is crucial for normal brain development.

**FIGURE 4:**
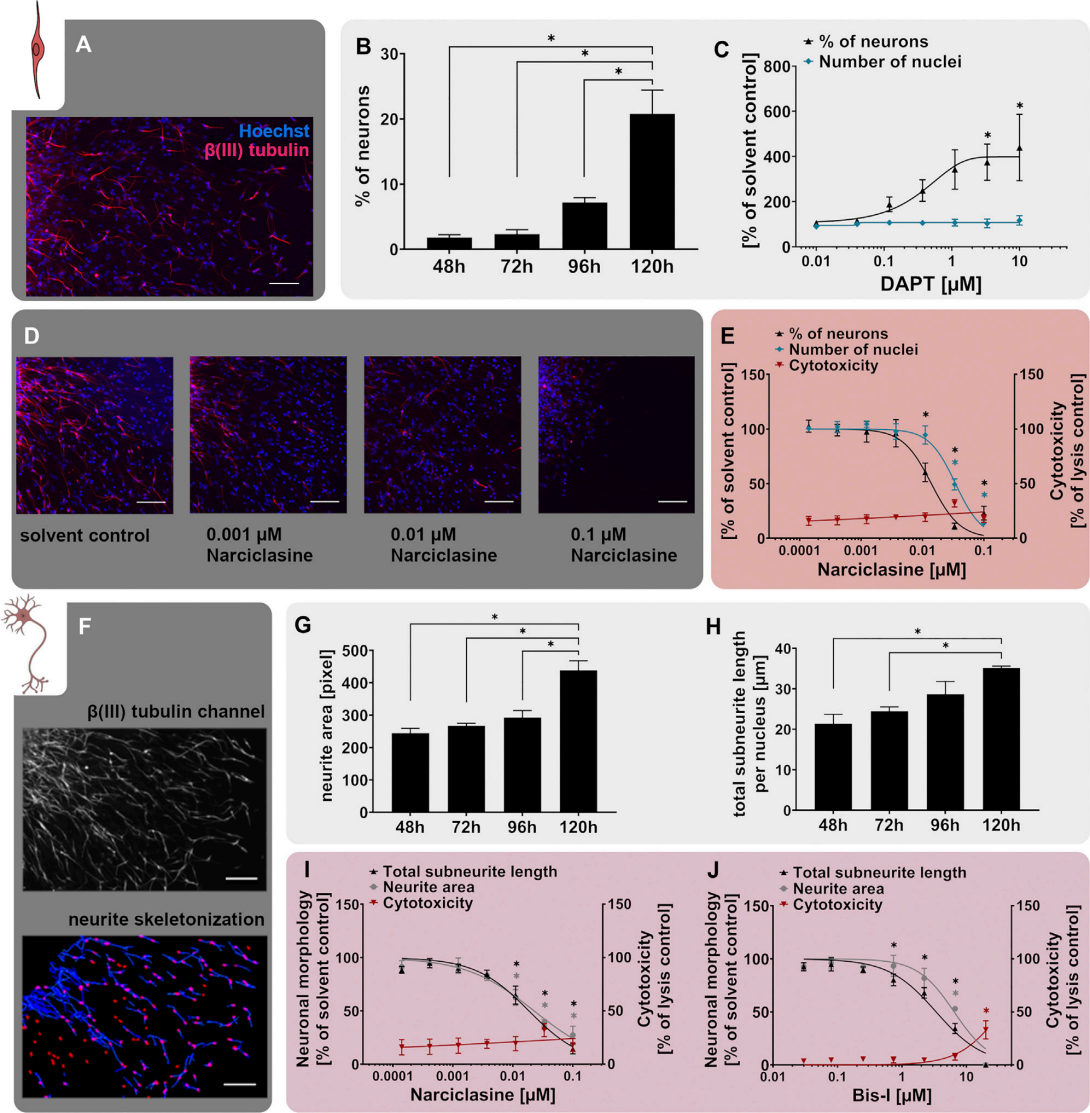
The NPC3 and NPC4 assays identify disruption of neuronal differentiation and morphology. Primary hNPC neurospheres were differentiated on PDL-laminin-coated matrices for 5 days without growth factors. **(A)** Immunocytochemical stainings for β(III)tubulin (neurons, red) and Hoechst33258 (nuclei, blue) confirmed neuronal marker expression and morphology. Scale bar: 100 µm. **(B)** Neuronal differentiation, assessed as the percentage of β(III)tubulin-positive neurons compared to the total nuclei count within the migration area, increased gradually over the 5 days of differentiation. **(C)** hNPCs differentiation for 5 days in presence of increasing concentrations of the Notch inhibitor DAPT (0.01 µM–10 µM) concentration-dependently decreased neuronal differentiation compared to the solvent control. **(D,E)** Treatment with the RhoA activator narciclasine (0.00014 µM–0.1 µM) for 5 days concentration-dependently decreased neuronal differentiation compared to the solvent control (SC). Representative pictures of β(III)tubulin- and Hoechst33258-stained cells **(D)** and concentration-response curves **(E)** are shown (adapted from Masjosthusmann et al. (2020). Scale bar: 100 µm. Cytotoxicity (LDH release) was assessed in parallel and is depicted as % of a lysis control (differentiated hNPCs treated with 0.2% Triton-X100). **(F)** The NPC4 assay detects the neuronal morphology of hNPC-derived β(III)tubulin-positive neurons. After the neurons were annotated by the convolutional neural network of the AI, neurite length and area were calculated by the Omnisphero software (Schmuck et al., 2017). **(G+H)** Neurite area and total subneurite length gradually increased over the 5 days of differentiation. **(I+J)** Both the RhoA activator narciclasine and the PKC inhibitor bisindolylmaleimide 1 (Bis-I) concentration-dependently decreased the neurite area and subneurite length compared to the respective solvent control in hNPCs differentiating over 5 days (adapted from Masjosthusmann et al., 2020). Cytotoxicity (LDH release) was assessed in parallel and is depicted as % of a lysis control (differentiated hNPCs treated with 0.2% Triton-X100). Data are presented as mean ± SEM. Statistical significance was calculated using one-way ANOVA. A *p*-value below 0.05 was termed significant. * significantly changed compared to the respective solvent control.

As a very simplified model, the Neurosphere Assay mimics cortex development *in vitro* since during the time-course of hNPC differentiation, neurons arise and migrate along the scaffold of RG cells (Fritsche et al., 2018b). Such young neurons are typically bipolar in shape and display neurites that show very limited branching (Figures 4A,E,F and Budday et al., 2015). Using high content imaging (HCI) and a subsequent artificial intelligence (AI; developed in collaboration with Prof. Dr. Axel Mosig (Ruhr University Bochum), Förster et al., 2021) we define neuronal identity in the mixed-culture neurosphere migration area due to immunocytochemical stainings with β(III)tubulin (Figure 4A). Over time, neurons progressively appear in the migration zone representing approximately 20% of the mixed culture after 5 days (Figure 4B), which is the time point of endpoint analysis in the NPC3 test method.

Neurogenesis during brain development is primarily regulated by the Notch signaling pathway, which is evolutionarily highly conserved and operates at many stages of human brain development (Yoon and Gaiano, 2005; Louvi and Artavanis-Tsakonas, 2006; Pierfelice et al., 2011). Stimulation of the Notch pathway could be correlated with impaired neuronal differentiation *in vivo* (Zhou W. et al., 2016; Zhang et al., 2018). In contrast, inhibited Notch signaling is known to accelerate neuronal differentiation *in vitro* and *in vivo* (Borghese et al., 2010). This can be pharmacologically excited by the Notch receptor inhibitor N-[N-(3,5-Difluorophenacetyl)-Lalanyl]-S-phenylglycine t-butyl ester (DAPT) through blockage of the presenilin-y-secretase complex (Dovey et al., 2001). Moreover, the Notch pathway is involved in the formation of long-term memory and is thus a putative actuator of developmental disorders (Costa et al., 2005). According to our comprehensive transcriptomic analysis, differentiating hNPCs express Notch receptors 1-3 (Masjosthusmann et al., 2018). Here we show that similar to our previously published work after 72 h of differentiation (Masjosthusmann et al., 2018), DAPT increases neuronal numbers to 187% and 439% of the respective solvent control at 0.12 and 10 µM DAPT, respectively, after 5 days of differentiation. Of note, the total cell number was not affected, indicating that the increase in neuronal numbers is at the expense of another cell type within the mixed culture (Figure 4C). The higher sensitivity of hNPC towards DAPT in this study is possibly due to the longer experimental time. Moreover, here we use different individuals compared to the previous study. These results indicate that the human-relevant Notch signaling pathway, which is one of the main drivers of neuronal differentiation *in vivo*, is also active in the hNPCs *in vitro*.

The positive effect of Notch inhibition on neuronal differentiation is thought to be - at least in part - attributed to suppression of the Rho GTPase RhoA (Peng et al., 2019). Consistent with this notion, narciclasine, an activator of RhoA, reduced neuronal differentiation of primary hNPCs cultured for 5 days in a concentration-dependent manner, together with a less sensitive reduction of the nuclei number (Figures 4D,E adapted from Masjosthusmann et al. (2020)). Increased RhoA activity correlated with reduced neuronal differentiation of murine neural stem cells and human iPSCs (Yang et al., 2016; Bogetofte et al., 2019). In contrast, inactivated RhoA signaling was sufficient to stimulate axon regeneration and recovery of hindlimb function after spinal cord injury in mice (Dergham et al., 2002) supporting the concept of RhoA activity as an inhibitory driver of neurogenesis.

Besides the generation of adequate neuronal numbers during neurogenesis, neuronal maturation, especially neurite outgrowth, and branching are equally important for the functional maturation of the CNS. Perturbations of which are assumed to be linked to neurodevelopmental disorders like autism spectrum disorder in humans (Zikopoulos and Barbas, 2010). The NPC4 assay measuring neurite outgrowth builds upon the neuronal differentiation assay (NPC3) and evaluates the neurite morphology within the multicellular differentiated neurosphere culture. After AI-based identification of β(III)tubulin^+^ neurons, their morphological features, e.g. neurite length and neurite area, are evaluated (Figure 4F) using the Omnisphero software (Schmuck et al., 2017). During the 5 days of hNPC differentiation, neurite maturation is characterized by an elongation of neurites and an increase in neurite area (Figures 4G+H).

Consistent with the above-mentioned effect of RhoA activation on neuronal differentiation (NPC3), narciclasine also reduced both neurite area and neurite length (Figure 4I adapted from Masjosthusmann et al. (2020)) within the NPC4 assay. This is in line with previous studies, reporting that narciclasine reduced neurite outgrowth via the Rho-associated protein kinase (ROCK) pathway in neurons differentiated from LUHMES human neuronal precursor cells (Krug et al., 2013). Moreover, a contactin-1 knock-down-dependent increase in RhoA activity caused morphological alterations in rat cortical neurons *in vivo* (Chen et al., 2018). Neurite outgrowth is further regulated by protein kinase C (PKC), a serine/threonine kinase, which controls various cellular responses by phosphorylation of substrate molecules and alteration of gene transcription (Nishizuka, 1986; Newton, 1995). While PKC activation induced neurite outgrowth in rat pheochromocytoma (PC-12) cells and primary rat spinal cord neurons from embryonic day 14 (Hundle et al., 1995; Yang et al., 2010), inhibition of PKC with the PKC inhibitor bisindolylmaleimide 1 (Bis-I) reduced neurite growth in PC-12 cells (Das et al., 2004), rat cortical neurons and human iPSC-derived neurons (Druwe et al., 2016). Similar effects were identified in the NPC4 assay upon exposure of hNPCs to Bis-I, which reduced neurite length and area compared to the solvent control (Figure 4J adapted from Masjosthusmann et al. (2020)).

Neuronal differentiation and maturation are tightly regulated processes, which are controlled by a variety of different signaling pathways, whose perturbation can cause severe adverse neurodevelopmental effects. The NPC3 (neuronal differentiation) and NPC4 (neuronal morphology) assays respond to known pathway modulators regulating neurogenesis and neurite outgrowth *in vivo* and are therefore predictive assays to identify chemicals disturbing neuronal development.

### 3.4 Oligodendrocyte Differentiation (NPC5)

**FIGURE 5:**
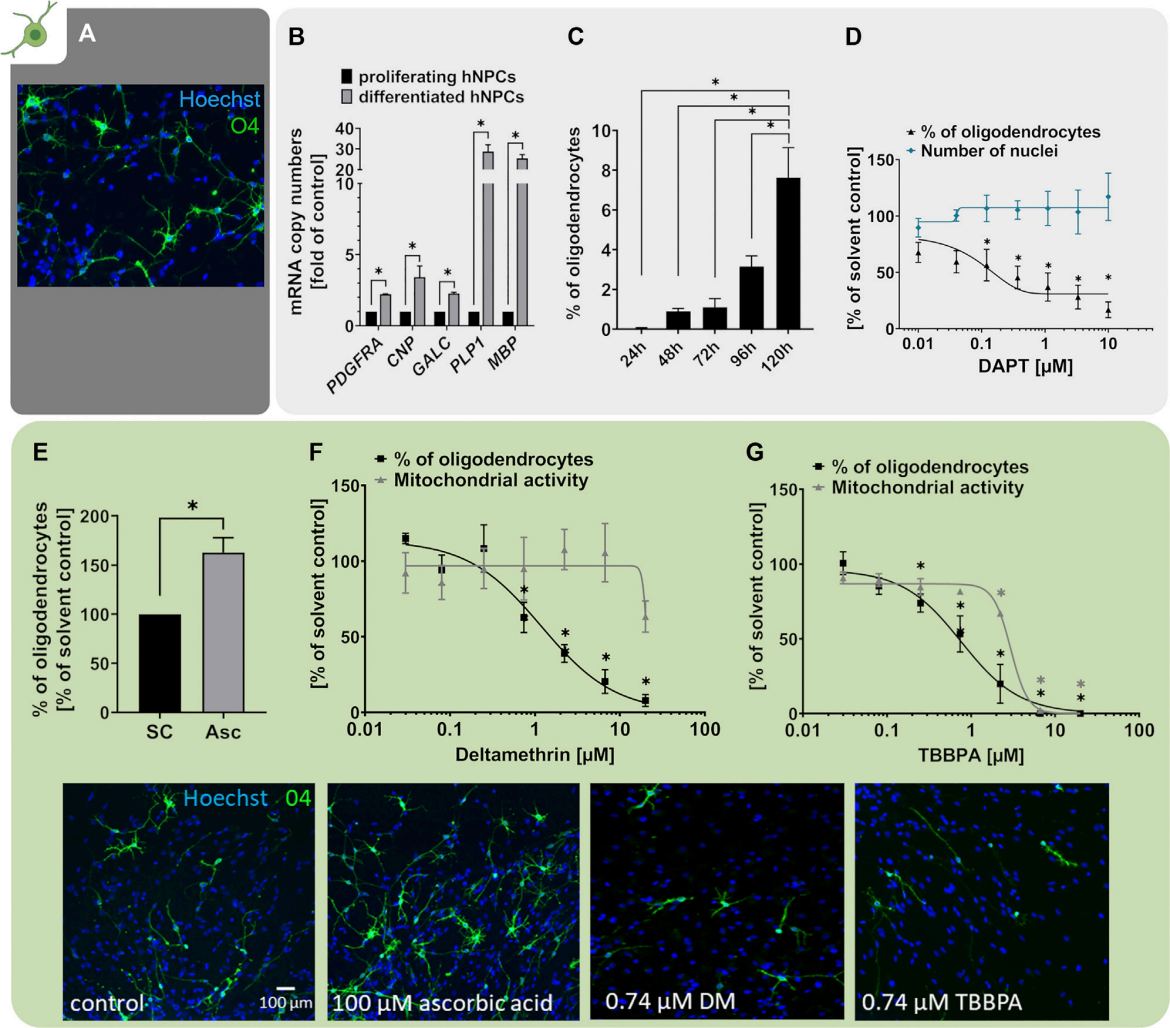
Oligodendrocyte differentiation is assessed with the NPC5 assay. **(A)** Primary hNPCs were differentiated for 5 days in differentiation medium without growth factors on PDL-laminin-coated plates. Immunocytochemical stainings were performed to identify cells of the OL lineage (O4) and cell nuclei (Hoechst33258). **(B)** mRNA expression of OL lineage markers *PDGFRA*, *CNP*, *GALC*, *PLP1* and *MBP* was assessed in proliferating hNPCs and hNPC differentiated for 60 h using quantitative real-time PCR. Expression was calculated as copy numbers (CN) per CN of the reference gene *ACTB* multiplied by 10.000. The expression in differentiated hNPCs is displayed as fold of expression in proliferating hNPCs. Expression of all markers increased during hNPC differentiation (adapted from Klose et al. (2021a)). **(C)** Oligodendrocyte differentiation, assessed as the percentage of O4-positive OLs compared to the total nuclei count within the migration area, increased gradually over the 5 days of differentiation. **(D)** hNPCs differentiation for 5 days in presence of increasing concentrations of the Notch inhibitor DAPT (0.01 µM–10 µM) concentration-dependently decreased OL differentiation compared to the solvent control. **(E)** hNPCs differentiation in presence of 100 µM ascorbic acid (Asc) increased the percentage of OLs in the differentiated culture. **(F+G)** Both exposure to the insecticide deltamethrin (DM, 0.027–20 µM, adapted from Masjosthusmann et al., 2020) and the flame retardant tetrabromobisphenol A (TBBPA, 0.027–20 µM, adapted from Klose et al., 2021a) during the 5 days of differentiation concentration-dependently decreased OL differentiation compared to the respective solvent controls. For deltamethrin and TBBPA the mitochondrial activity was assessed in parallel and is depicted as % of solvent control. For **(E–G)**, representative pictures of O4^+^ OLs exposed to solvent control or the respective treatments are shown. Data are presented as mean ± SEM. Statistical significance was calculated using one-way ANOVA **(C,D,F,G)** and two-tailed Student’s t-tests **(B,E)**. A *p*-value below 0.05 was termed significant. *significant compared to the respective solvent control.

Myelinating oligodendrocytes (OLs) are responsible for the formation of insulating myelin sheaths, thus accelerating the conduction of electrical impulses along axons and preserving axonal integrity during neurodevelopment and beyond. OLs derive from NPCs and RG cells differentiating into oligodendrocyte precursor cells (OPCs) and terminally into pre-myelinating OLs (pre-OLs) and myelin-producing mature OLs (Emery, 2010; van Tilborg et al., 2018). The OL-derived myelin ensheathing neuronal axons is indispensable for the development and function of the human brain (reviewed in Barateiro et al. (2016)). However, both pre-OLs and myelinating OLs are susceptible to various stressors including oxidative stress, astrogliosis, excitotoxicity and inflammation (reviewed in van Tilborg et al. (2016)) rendering them susceptible to a variety of exogenous stressors. Disturbances in oligodendrogenesis during neurodevelopment are associated with hypomyelination and white-matter deficits manifesting in clinical pathologies including the Allan-Herndon-Dudley Syndrome (Sarret et al., 2010) and periventricular leukomalacia (PVL; Back et al., 2001). Since the pool of OLs in humans remains stable after childhood, especially interference in OL development during the neurodevelopmental period is crucial (Yeung et al., 2014). The generation of pre-myelinating OLs can be modelled in hNPCs *in vitro* (NPC5). Differentiation of hNPCs over 5 days generated cells expressing the OL-marker O4, which exhibit the typical OL morphology with multiple branched processes necessary to ensheath neuronal axons (Figure 5A). Compared to undifferentiated hNPC neurospheres, differentiating hNPCs significantly increased mRNA expression of the OL markers *PDGFRA* (platelet-derived growth factor alpha, PDGFRα), *CNP* (CNPase), *GALC* (Galactosylceramidase), *PLP1* (proteolipid protein 1), and *MBP* (myelin basic protein) already after 60 h (Figure 5B adapted from Klose et al. (2021a)). While *PDGFRA* is predominantly a marker of immature OPCs, especially *MBP* is a myelin-associated gene increasingly expressed during oligodendrocyte maturation (Barateiro and Fernandes, 2014; Marinelli et al., 2016). Based on the marker expression and the highly branched morphology (Figures 5A,B), we conclude that our pre-OLs exhibit a certain degree of maturity. Similar to the neuronal differentiation described above, also the percentage of OLs within the multicellular hNPC-derived migration area increased over the differentiation time resulting in approximately 8% OLs after 5 days (Figure 5C; Moors et al., 2009).

It is well studied that several signaling pathways, including the Notch pathway, regulate NPC differentiation into OPCs (reviewed by He and Lu, 2013). A study on zebrafish embryos revealed that Notch is responsible for increased production of OPCs from ventral spinal cord precursors and that the increased OPC number is not due to increased OPC proliferation (Snyder et al., 2012). Moreover, contactin/F3-dependent Notch signaling promoted OPC differentiation from the rat oligodendroglial OLN-93 cell line and further increased the expression of myelin-associated glycoprotein (*MAG*; Hu et al., 2003). In line with that, differentiation of hNPCs in presence of the Notch inhibitor DAPT concentration-dependently decreased the percentage of O4^+^ cells compared to the solvent control, indicating that Notch signaling is a prerequisite for hNPC differentiation into the OL lineage (Figure 5D). In addition, OL differentiation is negatively influenced by bone morphogenic protein (BMP) 7 (Baumann et al., 2015) and BMP2 (Masjosthusmann et al., 2018), proteins of the transforming growth factor β family. BMP 2 and 7 also negatively regulated oligodendrocyte differentiation of primary rat NPC generated from E17 and PND2 brains (Zhu et al., 1999) and reduced myelin gene expression in Schwann cells (Liu et al., 2016). These data demonstrate that two major developmental pathways, i.e. Notch and BMP, are functional in these hNPCs.

Several studies — including observations in humans — confirmed that pre-OLs are especially susceptible to oxidative stress and that pre-OL damage by reactive oxygen species (ROS) is a potential underlying factor for the emergence of the cerebral white matter injury termed periventricular leukomalacia (PVL) (reviewed in Volpe et al. (2011)). In accordance, Guo et al. (2018) reported that the ROS scavenger vitamin C (ascorbic acid, Asc, 150 µM) enhanced the differentiation of primary mouse NPC-derived OPCs into OLs and further promoted expression of OL lineage markers O4, CNPase and MBP concentration-dependently (Guo et al., 2018). We also observed that ascorbic acid enhanced hNPC-derived OL maturation (Dach et al., 2017). However, in contrast to Guo et al. (2018), we did not observe this enhanced maturation in OLs derived from PND1 mouse neurospheres. This might be due to different developmental stages of animals as Guo et al. (2018) used cortices of E14.5 mouse embryos for NPC generation. Likewise, differentiation of hNPCs in presence of 100 µM ascorbic acid increased the percentage of O4^+^ oligodendrocytes within the NPC5 assay by approximately 60% (Figure 5E). This is in contrast to our previously published data where ascorbic acid solely induced maturity but not the number of OLs (Dach et al., 2017), which might be explained by inter-individual differences of the one individual used in the Dach et al. (2017) compared to the three individuals in this study.

Due to the particular sensitivity of OPCs and pre-OLs towards multiple stressors including ROS, excitotoxic damage, thyroid hormone disruption, or inflammatory cues (Volpe et al., 2011; Barateiro et al., 2016; Chesnut et al., 2021a), it is hypothesized that they might also be highly sensitive towards a variety of chemical noxae (Chesnut et al., 2021a). Within the NPC5 assay, we here show as two examples that both the insecticide deltamethrin (DM, Figure 5F adapted from Masjosthusmann et al. (2020)) and the organophosphate flame retardant tetrabromobisphenol A (TBBPA, Figure 5G adapted from Klose et al. (2021a)) diminished the number of O4^+^ oligodendrocytes concentration-dependently. Childhood exposure to pyrethroids like DM correlates with neurodevelopmental disorders including autism, attention deficit hyperactivity disorder (ADHD) and developmental delays reviewed in Pitzer et al. (2021). Likewise, developmental and early-life exposure to DM in rodents is associated with ADHD-like and anxiety-like behavior as well as deficits in working memory and spatial learning, often depending on the developmental stage of exposure (reviewed in Pitzer et al. (2019), (2021); Richardson et al. (2015)). The primary mode-of-action (MoA) of DM for its anti-pest action in mature neurons is the prolonged opening of voltage-gated sodium channels (VGSC). OPCs express active VGSC rendering this MoA highly likely for DM action on this immature OL state (comprehensively summarized in Hernández-Jerez et al. (2021)). In addition, DM induces oxidative stress and lipid peroxidation, which most likely also contribute to its neurotoxicity (reviewed in Pitzer et al. (2021)). The flame retardant (FR) TBBPA interferes with brain development in rodents (Hendriks et al., 2015; Rock et al., 2019) and its DNT-relevance for humans is supported by studies reporting bioaccumulation in maternal serum, cord blood and breast milk (Cariou et al., 2008; Kim and Oh, 2014). The adverse effects of TBBPA on OL differentiation in the NPC5 assay were accompanied by deregulation of a gene cluster involved in cholesterol metabolism suggesting lipotoxicity as TBBPA’s MoA (Klose et al., 2021b). Since myelinating OLs exhibit an exceptionally high rate of cholesterol metabolism, disturbances are particularly problematic in this cell type (Haq et al., 2003; Bezine et al., 2017).

In the past, the NPC5 assay has been identifying compounds of various substance classes as disruptors of OL differentiation including brominated as well as alternative organophosphate FRs (Dach et al., 2017; Klose et al., 2021a), sodium arsenite (Masjosthusmann et al., 2019), and a variety of substances within a recent screening project where the NPC5 assay was the most frequently positive assay across the Neurosphere Assay battery (Masjosthusmann et al., 2020). In neurotoxicological studies, oligodendrocytes are an understudied, yet highly relevant cell type that just recently received more attention (Chesnut et al., 2021a).

### 3.5 Thyroid Hormone (TH)-dependent Oligodendrocyte Maturation (NPC6)

In order to develop into myelinating OLs, OPCs and pre-OLs have to mature and express myelin-associated genes including myelin basic protein (MBP) and myelin proteolipid protein (PLP1). This maturation processes and the proper development of white matter tracts in humans depend on thyroid hormones (TH), such as the thyroxine metabolite triiodothyronine (T3; Annunziata et al., 1983; Baas et al., 1997; Murray and Dubois-Dalcq, 1997). In line with that, *in vivo* studies on hypothyroid rats reported reduced numbers of mature OLs and impaired expression of PLP1 and MBP (Ibarrola and Rodríguez-Peña, 1997; Schoonover et al., 2004). The devastating effects of TH disruption for human neurodevelopment are illustrated by clinical pathologies describing hypomyelination as a result of TH insufficiencies, including congenital hypothyroidism, maternal hypothyroidism, or the Allan-Herndon-Dudley syndrome (AHDS). These conditions feature clinical symptoms ranging from mild cognitive deficits to severe intellectual disabilities (Haddow et al., 1999; Rovet and Daneman, 2003; Sarret et al., 2010). ADHS is caused by inactivating mutations in the monocarboxylate transporter 8 (MCT8), a TH transporter, which is responsible for TH transport into the brain, and thus required for OL maturation (Vatine et al., 2021).

**FIGURE 6:**
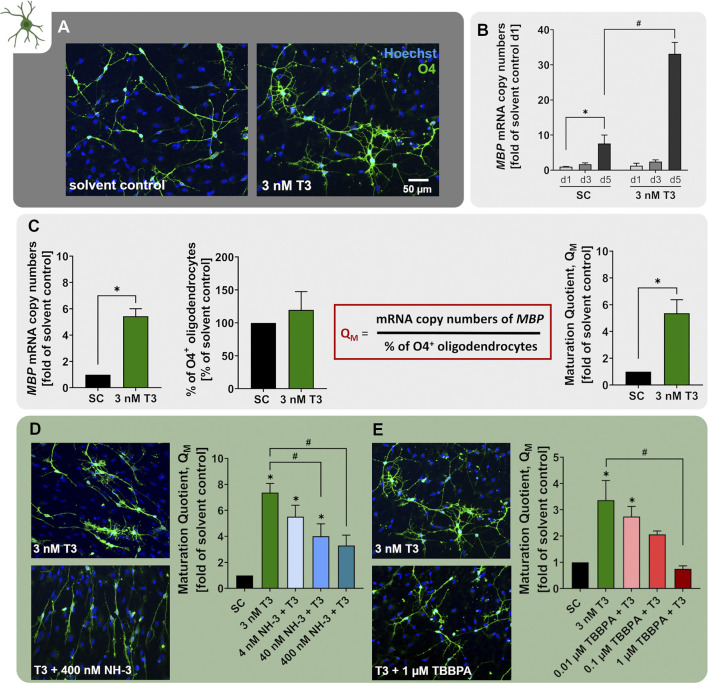
The NPC6 assay identifies disruptors of TH-dependent oligodendrocyte maturation. **(A)** Primary hNPCs were differentiated for 5 days in differentiation medium without growth factors on PDL-laminin coated plates either in presence of 3 nM T3 or solvent control. Immunocytochemical stainings were performed to identify cells of the OL lineage (O4) and cell nuclei (Hoechst33258). **(B)** mRNA expression of the OL lineage marker *MBP* was assessed in hNPCs differentiated in the presence of 3 nM T3 or solvent for 24, 72 or 120 h using quantitative real-time PCR. Expression is displayed as *MBP* mRNA copy numbers (CN) per CN of the reference gene ACTB multiplied by 10.000. The expression is displayed as fold of expression after 24 h (adapted from Dach et al., 2017). **(C)** OL maturation was quantified using the maturation quotient (Q_M_), which is calculated by dividing the *MPB* mRNA CN ((copy number *MBP*/ copy number *ACTB*) *10.000) by the percentage of O4^+^ cells. Exposure to 3 nM T3 significantly increased the Q_M_ compared to the solvent control (SC). **(D+E)** The Q_M_ was calculated for hNPCs differentiating for 5 days in presence of solvent (SC), 3 nM T3 alone or T3 in combination with increasing concentrations of the TH receptor antagonist NH-3 (4–400 nM) or the flame retardant TBBPA (0.01–1 µM). Both NH-3 and TBBPA concentration-dependently decreased the Q_M_ compared to 3 nM T3 and thus impaired T3-depedent OL maturation (adapted from Klose et al. (2021b)). Data are presented as mean ± SEM. Statistical significance was calculated using one-way ANOVA **(B, D, E)** and two-tailed Student’s t-tests **(C)**. A *p*-value below 0.05 was termed significant. *significant compared to the respective solvent control. ^#^significant compared to 3 nM T3.

Maturation of pre-OLs can be induced *in vitro* by differentiation of hNPCs in presence of 3 nM T3 (NPC6). Exposure to T3 clearly caused the O4^+^ cells to develop a more mature morphology with more branched processes (Figure 6A). Moreover, *MBP* mRNA expression increases over the time-course of differentiation already under control conditions, and even further in the presence of 3 nM T3 (Figure 6B adapted from Dach et al. (2017)). Since MBP is one of the major components of myelin, hNPC-derived pre-OLs differentiating in presence of T3 are on the path to myelinating OLs. In order to quantify the degree of OL maturation within the NPC6 assay, we calculated the maturation quotient (Q_M_), which is defined as the mRNA copy numbers of *MBP* per percentage of O4^+^ cells within the migration area. In line with the multitude of studies reporting that TH favor OL maturation (Baas et al., 1997; Dach et al., 2017; Klose et al., 2021b), we observed an increase of the Q_M_ upon exposure to 3 nM T3 (Figure 6C). The TH-dependent maturation of hNPC-derived OLs within the NPC6 assay further reacts to the synthetic antagonist NH-3 (Nguyen et al., 2002; Singh et al., 2016) since NH-3 concentration-dependently reduced the Q_M_ indicating that disruptors of TH receptor signaling can be identified with the NPC6 assay (Figure 6D adapted from Klose et al. (2021b), Dach et al. (2017)). A human-relevant disruptor of OL maturation identified within the NPC6 assay is TBBPA. At low concentrations, not yet affecting OL differentiation, TBBPA disturbs TH-dependent OL maturation, hence concentration-dependently reducing the Q_M_ in hNPCs differentiated in presence of 3 nM T3 (Figure 6E adapted from Klose et al. (2021b)). Impaired OL maturation is accompanied by alteration of TH-dependent genes, including *EGR1*, *IGFBP4*, *IL33* and *KLF9* (Klose et al., 2021b). These data provide the scientific basis for studying the disruption of TH-dependent oligodendrocyte maturation in differentiating hNPC. In the past, the NPC6 assay identified both BDE-99 and perfluorooctanoic acid (PFOA) not to be disruptors of human TH-dependent OL maturation, although BDE-99 reduced OL numbers (Dach et al., 2017; Klose et al., 2021b).

### 3.6 Human iPSC-derived hiNPC proliferation, migration and differentiation (hiNPC1+2a+3)

For 21st-century toxicity evaluation, hiPSCs have been strongly promoted as the basis for diverse test systems since they are of human origin, have unlimited availability and resemble different features of the desired target tissues very well (Wobus and Löser, 2011; Jennings, 2015; Csöbönyeiová et al., 2016; Fritsche et al., 2021). For brain tissues, one can generate hiPSC-derived neural progenitor cell (hiNPC) neurospheres, a relatively simple and easy to generate cell system (Sareen et al., 2014; Hofrichter et al., 2017; Kobolak et al., 2020). The hiNPCs have the ability to differentiate into neurons and astrocytes in secondary 3D (Sareen et al., 2014; Paşca et al., 2015; Zhou S. et al., 2016; Hofrichter et al., 2017; Nimtz et al., 2020; Soubannier et al., 2020) and 3D cultures (Pamies et al., 2017; Sloan et al., 2017; Marton et al., 2019; Chesnut et al., 2021b). For the generation of OLs, however, hiNPC differentiation time takes at least 8 weeks (Pamies et al., 2017) and is therefore not directly comparable to the primary neurospheres which produce OLs within 5 days of differentiation. Here we present data on hiNPC proliferation, migration and the differentiation potential.

**FIGURE 7:**
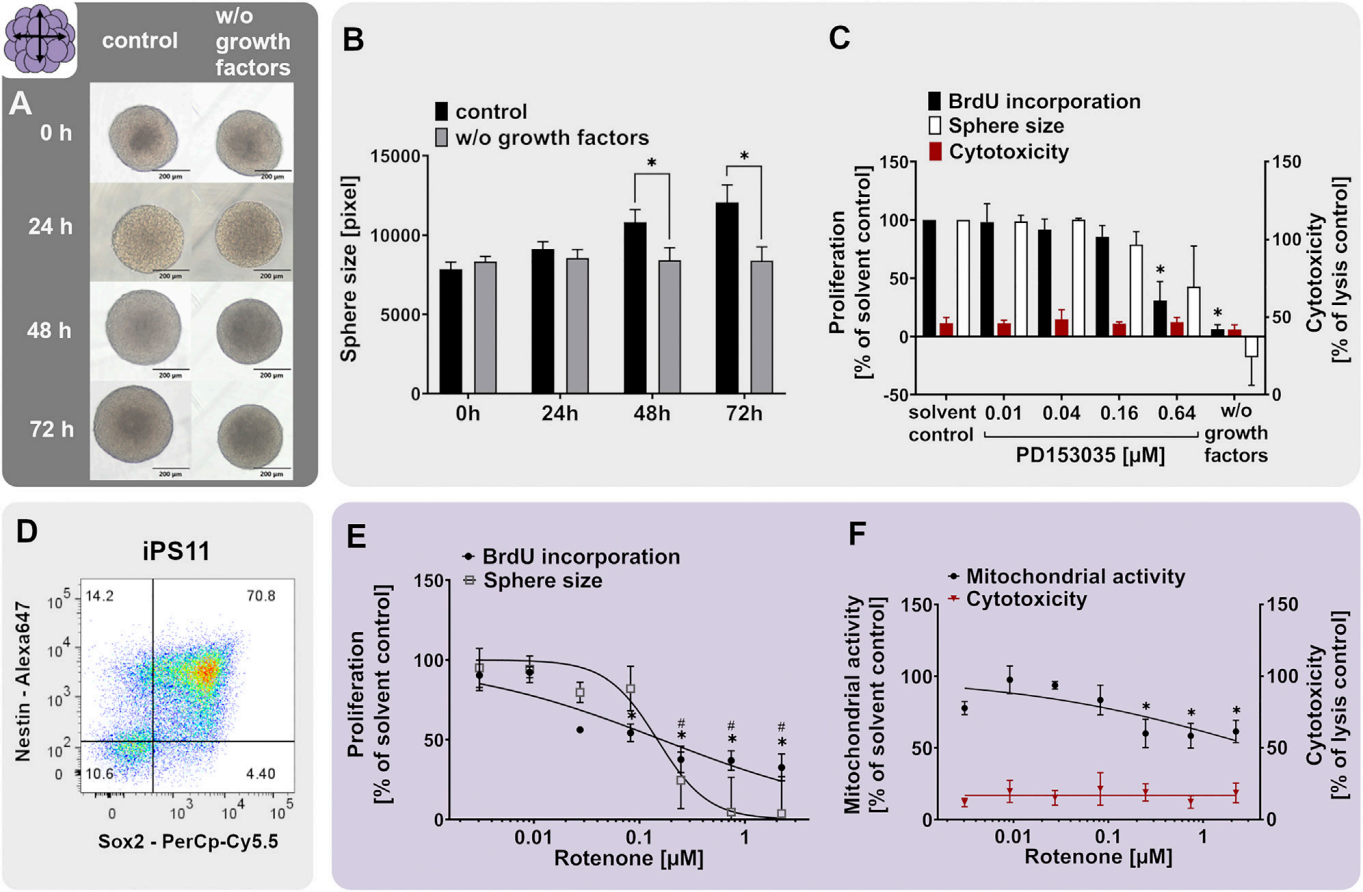
The proliferation of hiNPCs is assessed with the iNPC1ab assay. **(A,B)** Human iPSC-derived hiNPCs were cultivated for 3 days in proliferation medium containing 20 ng/ml of the growth factors EGF and FGF (control) or in medium without growth factors (w/o growth factors). Representative pictures **(A)** and quantifications of the sphere size **(B)**, as assessed within the iNPC1a assay, showed that growth factors are necessary for hiNPC proliferation. **(D)** Proliferating hiNPC neurospheres issued from an iPS11 neural induction were analyzed using flow cytometry analysis, confirming high expression of the neural stem/progenitor markers nestin and SOX2. The percentage of double-positive cells is indicated in the upper right quartile. **(C, E, F)** Exposure of proliferating hiNPCs for 3 days to increasing concentrations of the EGFR inhibitor PD153035 **(C)** or the mitochondrial complex I inhibitor rotenone **(E,F)** concentration-dependently decreased hiNPC proliferation compared to the respective solvent controls. Proliferation was assessed by sphere size increase (iNPC1a) and BrdU incorporation into the DNA (iNPC1b). The values of the chemical-treated conditions are expressed as % of the respective solvent controls. Cytotoxicity (LDH release) was assessed in parallel and is depicted as % of a lysis control (spheres treated with 0.2% Triton-X100). For rotenone-treatment, mitochondrial activity **(F)** was assessed in parallel and is depicted as % of solvent control. Data are presented as mean ± SEM. Statistical significance was calculated using one-way ANOVA **(C, E, F)** and two-tailed Student’s t-tests **(B)**. A *p*-value below 0.05 was termed significant. Symbols * and ^#^ show statistical differences in comparison to the solvent control of the respective endpoint if not marked otherwise.

When relating hiNPC (Figure 7A) to hNPC neurospheres (Figure 2A), they display the same neurosphere morphology and cannot be distinguished from each other with the bare eye. Moreover, hiNPCs express the neural stem/progenitor markers nestin and SOX2 (Figure 7D). The percentage of Nestin/SOX2 double-positive hiNPCs (70.8%) was in the same ballpark as that of hNPC (76.6%). Human iNPCs contained 10.6% cells not expressing any of the two markers, which is higher compared to the average of 1.9% of the three hNPC individuals (Figure 2). The proliferative capacity of hiNPCs was confirmed by measuring the sphere size increase over 3 days (iNPC1a) as shown in Figure 7B. hiNPC neurospheres increased their size by 53.6% during the 3 days in a proliferation medium containing EGF and FGF basic (control), whereas hiNPCs cultivated in proliferation media without growth factors (w/o growth factors) did not increase in size. In comparison, primary hNPC spheres only increased by approximately 30% in size over the 3 days (Figure 2B). EGF-dependent hiNPC proliferation is also EGFR-dependent since the EGFR inhibitor PD153035 decreased hiNPC proliferation (Figure 7C) similar to the primary hNPCs. These data demonstrate that the EGFR, as a crucial molecule for NPC proliferation, is also functioning in proliferating hiNPCs.

The proliferation of hiNPCs was also effectively inhibited by rotenone, an anti-proliferative compound with a known mode of action i.e. inhibition of the mitochondrial complex I of the electron transport chain (Saravanan et al., 2005). Rotenone produced oxidative stress in iPSC-derived neural stem cells (Pistollato et al., 2017) and mitochondrial dysfunction in human neural progenitor cells (Mahajan et al., 2019). In the present study, rotenone inhibited the proliferation of hiNPCs in a concentration-dependent manner with the lowest observed effect concentration of 30 nM (Figure 7E). In comparison, the proliferation of primary hNPCs was not affected by rotenone in the same concentration range (Masjosthusmann et al., 2020). Rotenone did not cause cytotoxicity in hiNPCs, however as expected from a mitochondrial complex I inhibitor, significantly reduced the mitochondrial activity (Figure 7F). Overall, the iNPC1ab assay behaves similar to the NPC1ab assay, however, hiNPCs proliferate faster.

**FIGURE 8:**
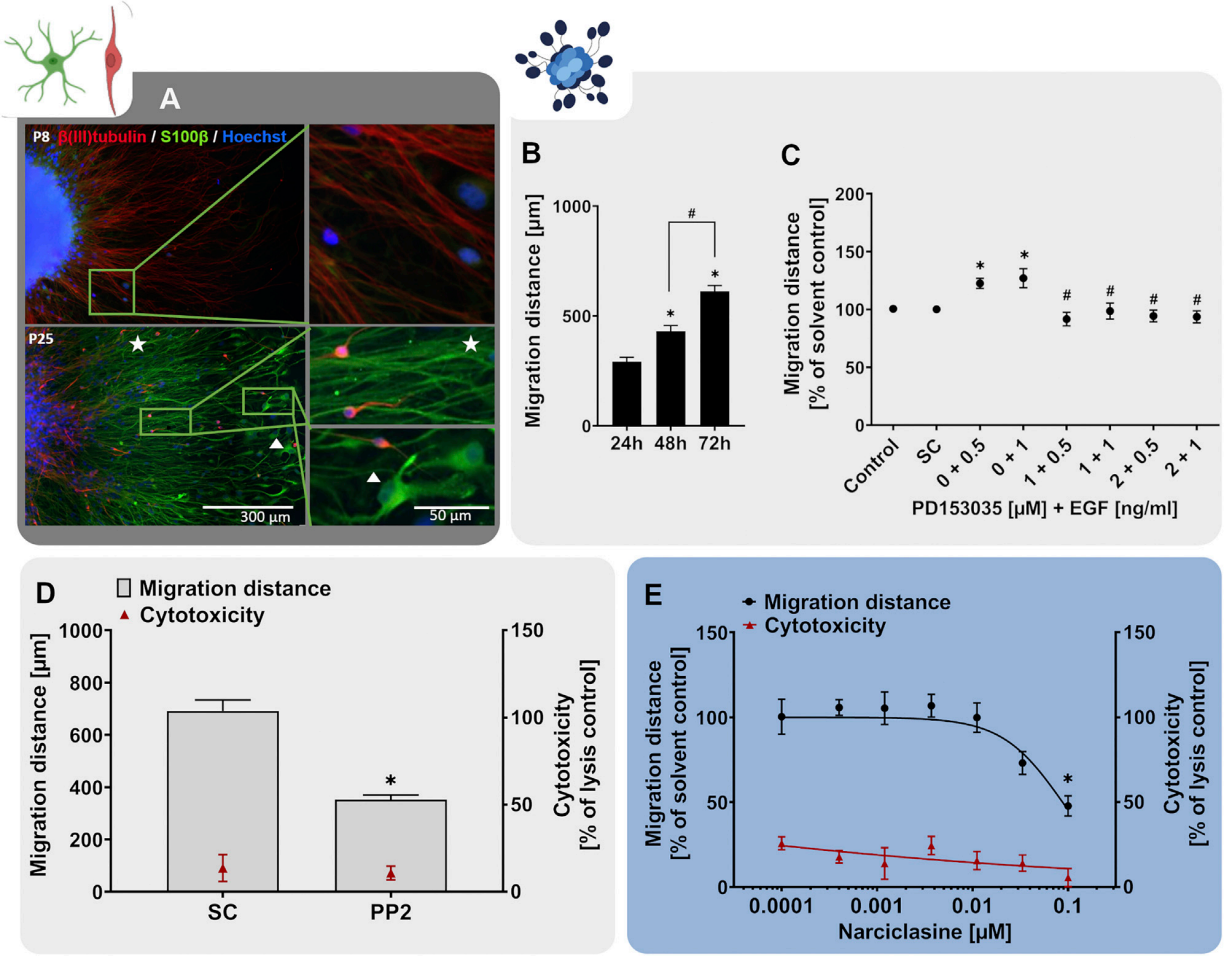
The hiNPC2 assay identifies disturbances of neural migration. Human hiNPCs were differentiated on PDL-laminin-coated 96-well plates in the absence of growth factors. **(A)** After 3 days of differentiation, immunocytochemical stainings of hiNPCs in early (p8) and later (p25) passages confirmed expression of the neuronal marker β(III)tubulin and the glial marker S100β. Nuclei were counterstained with Hoechst33258. Stars indicate RG-like cells and triangles indicate astrocyte-like morphology. **(B)** The migration distance of hiNPCs increased gradually over time, as assessed by determining the distance from the sphere core to the furthest migrated cells at four opposite positions in brightfield images every 24 h **(C)** hiNPC migration over 3 days was assessed in presence of EGF (0.5–1 ng/ml) alone, in combination with the EGFR inhibitor PD153035 (1–2 µM), or the respective solvent. While EGF increased hiNPC migration, PD153035 inhibited the EGF-induced effect. **(D)** A negative effect of the SRC-family kinase inhibitor PP2 on hiNPC migration was confirmed by differentiating hiNPCs for 3 days in presence of 10 µM PP2 or the respective solvent (SC). **(E)** hiNPCs differentiation in presence of increasing concentrations of narciclasine (MeHg, 0.0001–0.1 µM) for 3 days concentration-dependently reduced the migration distance. For **(D,E)**, cytotoxicity (LDH release) was assessed in parallel and is depicted as % of a lysis control (differentiated hiNPCs treated with 0.2% Triton-X100). Data are presented as mean ± SEM. Statistical significance was calculated using one-way ANOVA **(B, E)** and two-tailed Student’s t-tests **(C, D)**. A *p*-value below 0.05 was termed significant. *significant compared to the respective solvent control; ^#^significant compared to the respective EGF concentration.

Plating hiNPC neurospheres onto a PDL-laminin-coated matrix-initiated cell migration out of the sphere core accompanied by neuronal and astrocyte differentiation (Figure 8A). Importantly, the migration and neuron-glia cell differentiation patterns, as well as their respective cell morphologies highly depended on the sphere culture passage number. Early passages (P8) first and primarily differentiated into β(III)tubulin-positive neurons with elongated neurites that form dense neuronal networks followed by the appearance of S100β-positive astrocytes. Differentiation of hiNPCs from higher passages (P25) first led to the emergence of S100β-positive cells with RG-like morphology and subsequently of β(III)tubulin-positive neurons (Figure 8A). Regarding the S100β-positive cells, one could distinguish between elongated RG-like cells (Figure 8A, stars) and more star-shaped astrocytes (Figure 8A, triangle), the first being overrepresented in differentiating hiNPCs from higher passages.

Next, we inquired whether hiNPC neurospheres can also be used for studying neural migration. Therefore, we assessed the migratory capacity of hiNPC-derived cells (passages >17). After plating on PDL-laminin-coated matrices, the hiNPC-derived cells formed a circular migration area comparable to the primary hNPCs. Moreover, as observed for the hNPCs (Figure 3B), the migration distance of cells emerging from hiNPCs increased over time (Figure 8B). After 72 h, hiNPC migration was shorter (∼600–750 µm) than hNPC migration (∼950 µm, Figure 2), yet reproducible and fully sufficient for analyses.

As discussed above, EGFR-dependent signaling guides radial migration *in vivo* (Craig et al., 1996; Kornblum et al., 1998). To study migratory responses to EGF in hiNPCs, we measured the migration distances of hiNPC-derived cells in presence and absence of EGF and the EGFR inhibitor PD153035. Similar to hNPCs, the migration of plated hiNPCs was increased by both 0.5 and 1 ng/ml EGF, which was antagonized by co-treatment with PD153035 at concentrations of 1 and 2 µM (Figure 8C). Yet the EGF effects on hiNPC migration were weaker than in primary hNPCs (122% compared to 130% (0.5 ng/ml EGF) and 127% compared to 143% (1 ng/ml EGF)). This might be due to the developmental timing since the density of EGFR increases processivity through brain development (Burrows et al., 1997; Lillien and Raphael, 2000). Hence, the reduced responsiveness of hiNPCs to EGFR signaling could indicate that hiNPCs represent an earlier developmental time point compared to fetal hNPCs. However, since the observed differences were minor, additional studies are needed to thoroughly compare EGF function on hNPC and hiNPC migration. Further important regulators of neurodevelopmental migratory processes are SRC-kinases (Jossin et al., 2003; Kuo, 2005; Moors et al., 2007; Wang et al., 2015). Treatment of differentiating hiNPCs with the SRC-kinase inhibitor PP2 for 72 h reduced the migration distance to 51% of the respective solvent control without inducing any signs of cytotoxicity (Figure 8D). This is comparable to the hNPC response to PP2 exposure (Figure 3D). Last, we studied the effects of the RhoA GTPase activator narciclasine on hiNPC migration. RhoA activation reduced hiNPC migration in a concentration-dependent manner (Figure 8E). Comparing the hiNPC results (Figure 8E) to the response of primary hNPCs to narciclasine exposure (Masjosthusmann et al., 2020), the two cell systems did not differ in sensitivity (BMR_10_ 0.010 and 0.018 µM, respectively). In line with our observations, knockout of RhoA destabilized the actin and tubulin cytoskeleton in neurons and especially in radial glia cells, resulting in accelerated migration *in vitro* and *in vivo* (Cappello et al., 2012). Hence, narciclasine-mediated activation of RhoA could cause a hyperstabilization of the cytoskeleton and thus impair migration. This was observed in PARK2 knockout hiPSC-derived neurons, where migration was reduced by RhoA upregulation and rescued by RhoA inhibition (Bogetofte et al., 2019). Likewise, methylmercury, a metal disturbing neural migration in humans, affected hiNPC and hNPC migration at similar concentrations (Hofrichter et al., 2017).

Taken together, hiNPC proliferation and migration (iNPC1/2) work similarly as in primary hNPCs (NPC1/2). More work is needed to understand if these two test systems have also distinct applicability domains, i.e. concerning developmental timing, or if these are redundant assays. Nevertheless, primary NPCs produce oligodendrocytes within a very short time of 5 days, whereas hiPSCs need several weeks to produce oligodendrocytes. In addition, the convolutional neuronal networks were trained to quantify neuron and oligodendrocyte differentiation in the primary NPC assays. This has not been established for differentiating hiNPC, hence objective quantification methods for cell differentiation are lacking for this test system. Therefore, the primary neurosphere assay possesses its unique selling points.

### 3.7 Applications of the Neurosphere Assay

The Neurosphere test methods, which allow studying a large variety of neurodevelopmental KE, are suitable for many different applications ranging from basic scientific to different regulatory questions. The Neurosphere Assay can be applied in low to medium throughput formats by manual pipetting up to larger-scale applications for screening purposes using liquid handling systems. In the past, we studied the contribution of a variety of signaling pathways including interleukin-7 (Moors et al., 2009), the extracellular related kinase Erk1/2 (Moors et al., 2007), NO signaling (Tegenge et al., 2011), BMP2, the EGFR in intrinsic signaling, Notch1 (Masjosthusmann et al., 2018) and TH signaling (Dach et al., 2017) on neurodevelopmental KE using the Neurosphere assay. Moreover, we assessed the effects of a large variety of compounds on the Neurosphere Assay KEs and studied their MoA for some of them (Fritsche et al., 2005; Moors et al., 2007; Schreiber et al., 2010; Gassmann et al., 2010, 2014; Baumann et al., 2016; Barenys et al., 2017; Masjosthusmann et al., 2019, 2020; Klose et al., 2021b, 2021a). In addition, species aspects were investigated using time-matched (Clancy et al., 2007) rat, mouse or rabbit neurospheres (Gassmann et al., 2010; Baumann et al., 2016; Barenys et al., 2017, 2021; Dach et al., 2017; Masjosthusmann et al., 2018, 2019; Ali et al., 2019; Kühne et al., 2019; Klose et al., 2021b). On the regulatory side, data from the Neurosphere Assay was used for hazard characterization of deltamethrin and flufenacet building an IATA (Hernández-Jerez et al., 2021). Also, the application of screening and prioritization was served by the neurospheres studying banned and currently in use flame retardants (Klose et al., 2021a). Last, data from the Neurosphere Assay contributed to the establishment of putative AOPs (Bal-Price et al., 2015b; Barenys et al., 2020; Klose et al., 2021b) demonstrating the usefulness for helping to frame the regulatory landscape.

Currently, we are further expanding the future regulatory application of this promising test system. Firstly, we have been studying the contribution of 14 hormone receptors, i.e. AhR, RAR, RXR, GR, LXR, PPARα,δ/γ, TH, and the consequences of their disruption to hNPC development (Koch et al., in preparation) within the H2020 ENDpoiNTs project (Lupu et al., 2020). This work shall bring about new test methods for studying endocrine disruption-related DNT (ED-DNT) for regulatory application. Secondly, the Neurosphere Assays are used for feeding and substantiating ontologies for risk assessment purposes concerning cognitive function defects within the H2020 ONTOX project (Vinken et al., 2021). Moreover, more radial and astroglia-related endpoints are currently established with the hNPC test system, since these cell types are not entirely covered in the current assay setup. Besides these applications, we are currently enlarging the data basis for signaling pathways known to be crucial for human brain development (Fritsche, 2017; Sachana et al., 2021b). Altogether these data will continue to define the biological and toxicological applicability domains of the Neurosphere Assay and hence increase confidence in this valuable assay.

To use the results from the Neurosphere Assay in a risk assessment context, the calculated Point of Departure (PoD) values, in our case a benchmark concentration (BMC), need to be translated to an internal dose within the fetal brain. Therefore, reverse physiology-based kinetic modeling (PBK) and quantitative *in vitro* to *in vivo* extrapolations (qIVIVE) can be applied (Basketter et al., 2012; Proença et al., 2021). One fundamental input to IVIVE is the determination of the free test compound concentration, which is defined as the concentration of the compound not bound to plastics, protein or lipid. If the nominal concentration is used instead, the uncertainty of the data analysis increases. The internal dose, determined by qIVIVE can then be translated into an external dose which highly depends on the exposure scenario (e.g. oral, dermal) and modeling of the barriers relevant for the respective type of exposure (e.g. oral bioavailability, dermal bioavailability). Especially in the DNT-context not only the classical parameters like uptake, distribution, metabolism and excretion are relevant, but additional modeling of the blood-placenta barrier and the developing blood-brain-barrier is of the highest relevance. The calculated external concentration can then be used to determine a toxic threshold dose and define an acceptable daily exposure/intake. As an alternative to the approach, known human exposures can be used as a starting point. As an example, they can be modeled from average food intake, dermal exposure or measured as plasma concentrations in epidemiological studies (Sexton et al., 1995). Moreover, exposure limits from animal studies that evidently caused a DNT phenotype can be used as a starting point for the setup of *in vitro* experiments. Another important point that needs to be considered is the metabolism of the test compound in the human body, which is only partially covered in a human cell-based assay. To some extent enzymes are present in the different cell types which metabolize the test compound, however, the complete liver metabolism is absent. Therefore, the metabolism and distribution of a test compound have to be modeled using PBK and *in vitro* screening of metabolites instead of parent compounds has to be considered depending on the distribution of the parent compound and metabolites *in vivo*. In a neurodevelopmental context, PBK modeling could be used in the future to calculate fetal brain concentrations from plasma concentrations measured *in vivo*. These values can then be compared to the PoD values determined with the Neurosphere Assay.

## 4 Conclusion

The scientific validation of the Neurosphere Assay demonstrates that the neurodevelopmental processes, i.e. NPC proliferation, migration, neuronal differentiation, neurite outgrowth, oligodendrocyte differentiation and maturation, are well represented by the test methods. However, they denote a certain developmental time, the fetal period, and during this time especially early neurodevelopmental processes, like the switch from proliferation to initial migration and differentiation. However, how the assays are set up at the moment using an *in vitro* time of only 5 days, the developmental KEs are not followed to full cell maturity. Neurons stay in a mostly bipolar state and also oligodendrocytes do not reach the full myelinating condition. For studying earlier time-points during development, i.e. the embryonic period, or more mature endpoints, i.e. neuronal network formation and function or neuronal myelination, other assays are necessary. The current DNT IVB is evolving to close such biological gaps, yet thorough scientific validation has to be a prerequisite for each novel test system/method to proceed from hazard characterization finally to contributing to risk assessment for DNT using NAMs. Our data provide the rationale for the scientific validity of the endpoints depicted with the Neurosphere Assays. Hence, the DNT IVB, with the Neurosphere Assay as an integral part, is on a solid way to regulatory acceptance.

## Data Availability

The raw data supporting the conclusions of this article will be made available by the authors upon request.
